# Previously claimed male germline stem cells from porcine testis are actually progenitor Leydig cells

**DOI:** 10.1186/s13287-018-0931-0

**Published:** 2018-07-18

**Authors:** Yinshan Bai, Cui Zhu, Meiying Feng, Hengxi Wei, Li Li, Xiuchun Tian, Zhihong Zhao, Shanshan Liu, Ningfang Ma, Xianwei Zhang, Ruyi Shi, Chao Fu, Zhenfang Wu, Shouquan Zhang

**Affiliations:** 1 National Engineering Research Center for Breeding Swine Industry, Guangdong Provincial Key Lab of Agro-Animal Genomics and Molecular Breeding, College of Animal Science, South China Agricultural University, 483 Wushan Road, Tianhe District, Guangzhou, 510642 China; 2 School of Life Science and Engineering, Foshan University, Foshan, 528231 China; 3 Center for Regenerative Biology, Department of Animal Science, University of Connecticut, 1390 Storrs Road, Storrs, CT 06269 USA; 4 Department of Histology and Embryology, School of Basic Medical Sciences, Guangzhou Medical University, Guangzhou, 511436 China; 5 Key Laboratory of Cellular Physiology, Ministry of Education, Department of Cell Biology and Genetics, Shanxi Medical University, Taiyuan, 030001 China

**Keywords:** Male germline stem cells, Progenitor Leydig cells, Testicular cells, Gene expression, Pig

## Abstract

**Background:**

Male germline stem cells (mGSCs) offer great promise in regenerative medicine and animal breeding due to their capacity to maintain self-renewal and to transmit genetic information to the next generation following spermatogenesis. Human testis-derived embryonic stem cell-like cells have been shown to possess potential of mesenchymal progenitors, but there remains confusion about the characteristics and origin of porcine testis-derived stem cells.

**Methods:**

Porcine testis-derived stem cells were obtained from primary testicular cultures of 5-day old piglets, and selectively expanded using culture conditions for long-term culture and induction differentiation. The stem cell properties of porcine testis-derived stem cells were subsequently assessed by determining the expression of pluripotency-associated markers, alkaline phosphatase (AP) activity, and capacity for sperm and multilineage differentiation in vitro. The gene expression profile was compared via microarray analysis.

**Results:**

We identified two different types of testis-derived stem cells (termed as C1 and C2 here) during porcine testicular cell culture. The gene expression microarray analysis showed that the transcriptome profile of C1 and C2 differed significantly from each other. The C1 appeared to be morphologically similar to the previously described mouse mGSCs, expressed pluripotency- and germ cell-associated markers, maintained the paternal imprinted pattern of *H19*, displayed alkaline phosphatase activity, and could differentiate into sperm. Together, these data suggest that C1 represent the porcine mGSC population. Conversely, the C2 appeared similar to the previously described porcine mGSCs with three-dimensional morphology, abundantly expressed Leydig cell lineage and mesenchymal cell-specific markers, and could differentiate into testosterone-producing Leydig cells, suggesting that they are progenitor Leydig cells (PLCs).

**Conclusion:**

Collectively, we have established the expected characteristics and markers of authentic porcine mGSCs (C1). We found for the first time that, the C2, equivalent to previously claimed porcine mGSCs, are actually progenitor Leydig cells (PLCs). These findings provide new insights into the discrepancies among previous reports and future identification and analyses of testis-derived stem cells.

**Electronic supplementary material:**

The online version of this article (10.1186/s13287-018-0931-0) contains supplementary material, which is available to authorized users.

## Background

Male germline stem cells (mGSCs), localized to the basal lamina of seminiferous tubules, are a special kind of adult stem cell because they have the capacity to maintain self-renewal and to transmit genetic information to the next generation following spermatogenesis. By epigenetic reprogramming, mGSCs can be reprogrammed to become embryonic stem (ES)-like cells, termed as multipotent adult germline stem cells [1]. Although multipotent adult GSCs have been convincingly generated from mouse testis-derived mGSCs, under specific culture conditions without any genetic manipulation [2–5], similar attempts from human, monkey, and other species including pig have remained inconsistent and controversial [6, 7]. Evidence indicates that human testis-derived ES-like cells (termed haGSCs), with morphology similar to human pluripotent stem cells, are actually testis-derived multipotent mesenchymal stem cells [7–9]. Moreover, mouse mGSCs are believed to have an irregular grape-shaped morphology [10], whereas mGSCs of human and domestic animals have been claimed to show a regular three-dimensional morphology [11–13], or display fibroblast-like morphology [14, 15].

The porcine mGSCs represents as an ideal model for human reproduction and stem cell research [16]. Although previous studies have indicated that the porcine testis-derived stem cells show a three-dimensional morphology similar to haGSCs, they are still referred to as porcine mGSCs based on their expression of specific germline genes [11]. The appropriate identification markers and long-term culture systems for porcine mGSCs have not been established yet [6, 7]. Thus, confusion remains about the characteristics and origin of porcine testis-derived stem cells. Whether porcine mGSCs are actually of mesenchymal origin as demonstrated in haGSCs remain unknown.

Here, we have identified two types of testis-derived stem cells from our porcine testicular cell culture: mGSCs and progenitor Leydig cells (PLCs). The porcine mGSCs possess characteristics similar to those of mouse mGSCs, but are distinct from those of previously identified pig mGSCs. We show for the first time that, another distinct population of porcine testis-derived stem cells showing three-dimensional morphologies similar to those of the previously recognized porcine mGSCs, are in fact porcine PLCs.

## Methods

### Testes collection and testicular cell preparation

Testes from 5-day-old piglets (Landrace) were provided by Guangdong Wens Foodstuff Co., Ltd. (Yunfu, China). The porcine testicular cells were prepared and cultured as previously described [17]. The unattached testicular cells were gently transferred into a new 10-cm culture dish by differential plating culture, repeated hourly for five transfers. When the majority of the remaining suspended cells were putative porcine mGSCs, density gradient centrifugation on Percoll (Pharmacia) was performed as previously described [17]. After centrifugation, lymphocytes and cellular debris were in the top layer, erythrocytes were in the bottom layer, and the target cells were in the middle layer. The target cells were collected by centrifugation at 178×*g* for 3 min, and cultured for another 10 h to allow adhesion of non-germline stem cells including Sertoli cells, peritubular myoid cells and Leydig cells before obtaining a purified population of putative porcine mGSCs.

### In vitro culture and differentiation of porcine mGSCs

The purified putative porcine mGSCs were cultured on mouse embryonic fibroblast feeder cells treated with mitomycin C in a modified StemPro-34 SFM medium (Invitrogen), supplemented with 20 ng/mL recombinant rat glial cell line-derived neurotrophic factor (GDNF, R&D Systems), 10 ng/mL recombinant human basic fibroblast growth factor (bFGF, Peprotech), 10 ng/mL mouse epidermal growth factor (EGF) (Prospec), 1000 U/mL recombinant mouse leukemia inhibitory factor (LIF) (Millipore), 1 mM L-glutamine, 1% sodium pyruvate, 1% insulin-transferrin-selenium (ITS, Gibco), B27 supplements (Gibco), 5 μM pifitrin-α (Stem cell Technologies), 55 μM β-mercaptoethanol (β-ME, Gibco), 1% bovine serum albumin (BSA), 1% fetal bovine serum (FBS, Gibco), and 1% penicillin and streptomycin.

For induction of sperm differentiation, the cells were cultured in Dulbecco’s modified Eagle medium (DMEM) supplemented with 10% FBS, 500 ng/mL follicle-stimulating hormone, 5 μM vitamin A, 0.1 mM testosterone, 1% ITS, 1 mM L-glutamine, 1 mM sodium pyruvate, and 1% nonessential amino acid (Gibco) on porcine testicular fibroblasts as feeder cells.

### In vitro culture of porcine-induced pluripotent stem cells (piPSCs)

The piPSCs were cultured as previously described [18] in mTeSR™1 medium (Stem cell Technologies) supplemented with 1 mM L-glutamine, 1% ITS, 1% nucleosides (Millipore), 55 μM β-ME, and 1000 U/mL LIF, and the culture dishes were coated with 100 μg/mL poly-L-lysine.

### In vitro propagation and differentiation of porcine PLCs

The PLCs were cultured in DMEM medium supplemented with 15% FBS, 1% ITS, 1 mM L-glutamine, 1% nucleosides, 55 μM β-ME, 1000 U/mL LIF, 10 ng/mL EGF, and 20 ng/mL platelet-derived growth factor-BB (PDGF-BB, Peprotech). The PLC differentiation medium was supplemented with 15% FBS, 1% ITS, 1 mM L-glutamine, 1% nucleosides, 55 μM β-ME, 1:500 cholesterol, and 1 ng/mL luteinizing hormone (LH) (Peprotech).

### Immunohistochemistry and immunofluorescence

Testis tissue was fixed in 4% paraformaldehyde for 24 h and embedded in paraffin. The immunohistochemical analysis was performed as previously described [19]. The primary antibodies (Additional file 1: Table S1) against glial cell line-derived neurotrophic factor family receptor alpha-1 (GFRA1), octamer-binding transcription factor 4 (OCT4), and promyelocytic leukemia zinc finger (PLZF) were used for detecting porcine mGSCs, and with those against stem cell factor receptor (c-KIT), GATA binding protein 4 (GATA4); platelet-derived growth factor receptor A (PDGFRA1), LIF receptor (LIFR), NES gene (NESTIN), cytochrome P450 family 11 subfamily A member 1 (CYP11A1), cytochrome P450 family 17 subfamily A member 1 (CYP17A1), steroidogenic acute regulatory protein (StAR), and β hydroxysteroid dehydrogenase (3β-HSD) for detecting PLCs. The immunofluorescence analysis of the porcine testis-derived stem cells was performed as previously described [19].

### Analysis of alkaline phosphatase (AP) activity

The AP activity was performed using a commercial staining kit (Dingguo, Beijing, China) according to the manufacturer’s instructions as previously described [19]. Briefly, the cells were fixed in 4% paraformaldehyde for 20 min at room temperature, and washed three times with phosphate buffer saline (PBS). The detector reagents nitro blue tetrazolium and 5-bromo-4-chloro-3-indolyl-phosphate (NBT/BCIP) were then added for incubation at room temperature, in the dark, for 15 min. Finally, the reaction was terminated with three washes in PBS. The images were captured by light microscopy (Olympus).

### Analysis of flow cytometry

The putative porcine mGSCs or PLCs were fixed in 4% paraformaldehyde for 15 min. The fixed cells suspended in PBS containing 2% heat-inactivated FBS and 0.01% Triton X-100, were reacted for 2 h at 4 °C with primary antibodies (Additional file 1: Table S1) against GFRA1, OCT4, PLZF, and c-KIT to identify putative mGSCs, and CD29, CD44, CD45, CD51, CD73, and CD105 to identify putative PLCs. Then the cells were incubated with FITC-conjugated secondary antibodies for 30 min at 37 °C. The fluorescence intensity was analyzed by flow cytometry (BD Biosciences) and the FlowJo software (Version 10.0.7).

For cell cycle assay, cells were fixed with cold 70% ethanol, and incubated at 4 °C overnight. Fixed cells were centrifuged at 178×*g*, 4 °C for 3 min, and resuspended in 500 μL of PBS containing 50 μg propidium iodide (PI), 100 μg/mL RNase A, and 0.2% Triton X-100 at 37 °C for 30 min in the dark. For cell apoptosis assay, cells were trypsinized and centrifuged at 178×*g*, 4 °C for 5 min. Cell pellets were resuspended in 400 μL Annexin V binding buffer (BD Pharmingen™), and 5 μL Annexin V-FITC and 10 μL PI were added and incubated at room temperature for 15 min. Fluorescence of FITC and PI were measured by flow cytometry (Becton Dickinson FACStar Plus).

### RNA extraction and quantitative reverse transcriptase (RT)-PCR

Total RNA was extracted from cells using the RNeasy Mini Kit (Qiagen) according to the manufacturer’s instructions. Quantitative RT-PCR was performed using SYBR Green PCR Master Mix (Applied Biosystems) in an Applied Biosystems 7900HT Real-time PCR Thermo Cycler. The mRNA expression of targeted genes was normalized to *β-actin*. The primer sequences are given in Additional file 2: Table S2.

### Combined bisulfite sequencing analysis

Combined bisulfite sequencing analyses were performed as previously described [20], to investigate the promoter methylation levels of the pluripotent factors *Oct4* and *Nanog*, and the imprinted gene *H19*.

### Microarray analysis

High-throughput sequencing was performed using the Illumina HiSeq 3000 (Guangzhou RiboBio Co., Ltd., China). The RNA-seq was carried out with three biological replicates. RNA-seq data were uniquely mapped to susScr3 database by bowtie2-tophat2. Genes with absolute log 2-transformed fold changes > 2.0 were regarded as differentially expressed (DE) genes, and a threshold of *P* < 0.05 was used. The gene ontology (GO) analysis was performed by using DE genes against gene sets from the GO database (http://geneontology.org/) and Kyoto Encyclopedia of Genes and Genome (KEGG) pathway database (http://www.genome.jp/kegg/pathway.html).

### Differentiation ability of PLCs

Adipogenic differentiation was induced by sequentially culturing cells for 13 days, with 4 induction cycles of a 3-day culture with DMEM medium A and a 1-day culture with DMEM medium B, followed by detection with Oil red staining, as previously described [17].

Osteogenic differentiation was induced as previously described [21] with minor modifications. Briefly, the cells were cultured for 2 weeks with a differentiation induction medium consisting of DMEM with 10% FBS, 1 mM L-glutamine, 10 mM sodium glycerophosphate, 50 μg/mL vitamin C, and 1 nM dexamethasone. The osteogenic-differentiated cells were fixed in 4% paraformaldehyde, and the presence of calcium crystals was identified by Alizarin red staining.

The concentrations of testosterone were determined using a commercial ELISA kit (R&D systems) according to the manufacturer’s instructions.

### Collection, induced maturation and activation of oocytes

Ovaries were collected from a local slaughterhouse (Jiahe, Guangzhou, China) and transported at 37 °C within 1 h to our laboratory in 0.9% (*w*/*v*) NaCl solution supplemented with 1% penicillin and streptomycin. Cumulus-oocyte complexes (COCs) were obtained by aspiration from antral follicles (3–6 mm diameter) with a 12-gauge needle. The COCs were washed 3 times in Dulbecco’s Phosphate Buffered Saline (DPBS) supplemented with 4 mg/mL polyvinyl alcohol, and were matured at 38.5 °C in humidified atmosphere of 5% O_2_ and 5% CO_2_, as previously reported [20]. The mature oocytes were transferred to medium with 0.1% hyaluronidase, and the cumulus cells were removed by gentle aspiration with a pipette to get denuded metaphase II (MII) oocytes with a first polar body. Intracytoplasmic sperm injection (ICSI) was performed on a heated microscope stage at 38.5 °C as previously described [21].

After ICSI, oocytes were activated by a BTX Electro Cell Manipulator (Biotechnologies and Experimental Research, Inc., USA), with the parameters of 0.48 KV/cm and 30 μsec pulse at 1.26 KV/cm DC. Spermatozoa and induced sperm of C1 were collected for preparation of injection. Then activated oocytes were cultured with porcine zygote medium 3 (PZM-3) [20] in humidified atmosphere of 5% O_2_ and 5% CO_2_ at 38.5 °C.

### Statistical analysis

The data were analyzed using two-tailed Student’s t-test or one-way ANOVA by SPSS software (Version 19.0). Results are expressed as mean ± SEM. *P* < 0.05 was considered to be statistically significant.

## Results

### Isolation and characterization of putative porcine mGSCs

**Fig. 1:**
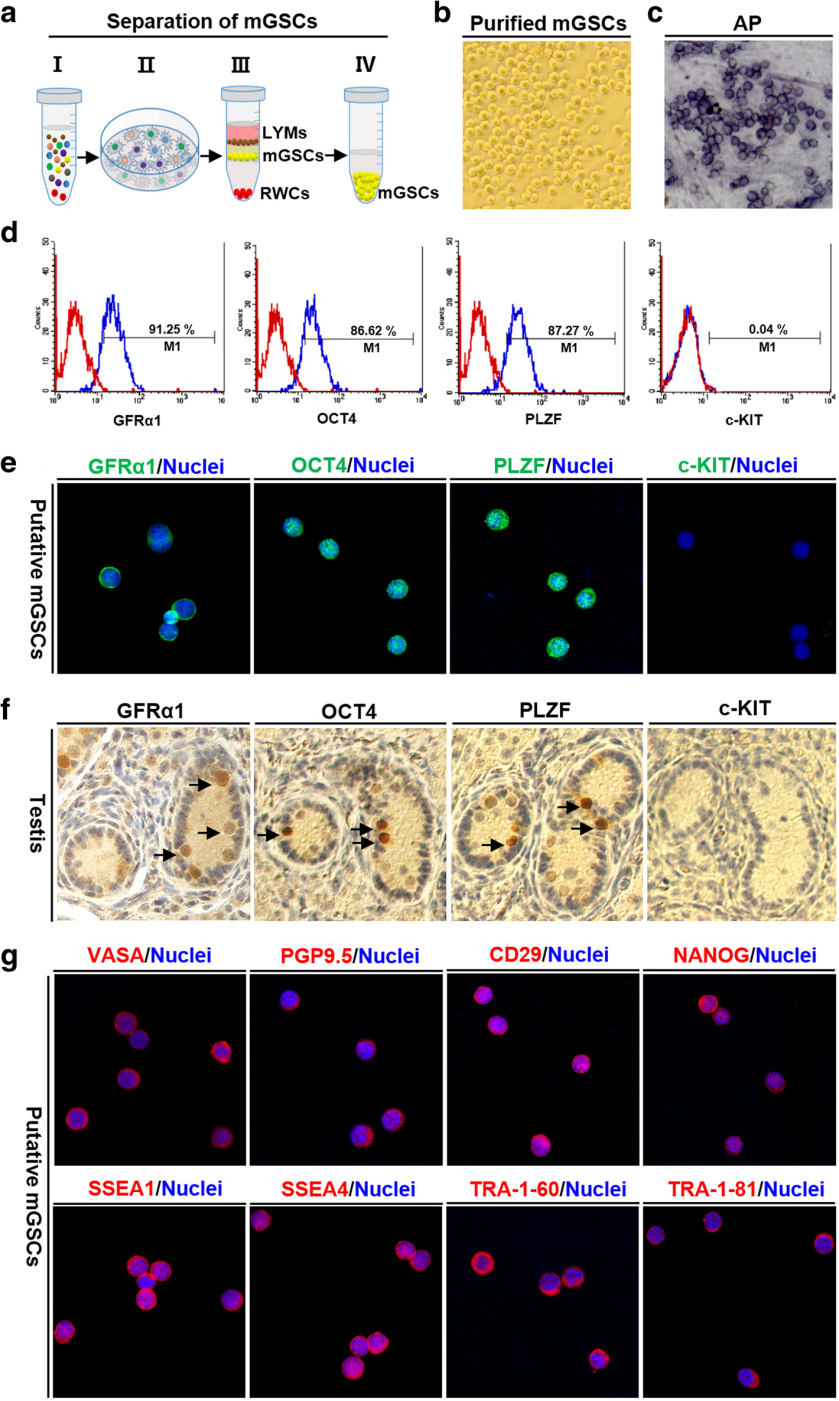
Isolation and characterization of putative porcine mGSCs from the testes. **a** A schematic illustrates the isolation method of putative porcine mGSCs from the testes of 5-day-old piglets. **b** The attached fibroblasts were removed from the cell suspension by differential plating culture, and an image shows the purified mGSCs. **c** An image shows the alkaline phosphatase (AP) staining of the isolated putative mGSCs. **d** The purity of putative isolated porcine mGSCs was demonstrated by flow cytometry with staining and analyses of GFRA1, OCT4, PLZF, and c-KIT. **e** Immunofluorescence staining shows expression of the stem cell markers GFRA1, OCT4, and PLZF in the putative porcine mGSCs; nuclei were stained with Hoechst 33,342 (blue) and c-KIT was used as negative control. **f** Immunohistochemistry staining shows expression of GFRA1, OCT4, PLZF, and c-KIT in the testis of a 5-day-old piglet. **g** Immunofluorescence staining shows expression of the crucial germ cell-associated proteins VASA, PGP9.5, and CD29 and the pluripotency-associated factors NANOG, SSEA1, SSEA4, TRA-1-60, and TRA-1-81 in putative porcine mGSCs

After two-step enzyme digestion and differential plating culture method (Fig. 1a), the majority of attached fibroblasts were removed, with retention of unattached cells, including putative mGSCs, leukomonocytes, and erythrocytes (Additional file 3: Figure S1). The Percoll density gradient centrifugation was then performed to remove leukomonocytes and erythrocytes (Fig. 1a). The putative purified porcine mGSCs showed a similar morphology to that of mouse mGSCs, with a high refractivity and nucleoplasm ratio (Fig. 1b and Additional file 3: Figure S1f), and displayed strong AP activity (Fig. 1c).

The purity of putative porcine mGSCs, were determined by analyzing expression of mGSC markers, including GFRA1, OCT4, and PLZF, which were 91.25%, 86.62%, and 87.27%, respectively. The negative control, differentiation marker c-KIT was not detected (Fig. 1d), which suggest that the isolated putative porcine mGSCs were quite pure and undifferentiated. Immunofluorescence staining showed that GFRA1 localized to the cell membrane, whereas OCT4 and PLZF were in the nuclei, of the putative porcine mGSCs (Fig. 1e), which indicated the presence of porcine mGSCs harvested from piglets of this same age (Fig. 1f).

Other crucial protein markers for germ cells, including DEAD-box polypeptide 4 (DDX4, also known as VASA), protein gene product 9.5 (PGP9.5), CD29, and NANOG, and membrane antigens for pluripotent stem cells, including stage-specific embryonic antigens 1 and 4 (SSEA1 and SSEA4), TRA-1-60, and TRA-1-81, were also expressed in the isolated putative porcine mGSCs (Fig. 1g). The marker VASA was localized to the basement membrane of seminiferous tubules, whereas the pluripotent factor NANOG was expressed in both the interstitial space and the seminiferous tubules of the testes from 5-day-old piglets (Additional file 4: Figure S2).

### Generation of two types of stem cell clusters from porcine testicular cell culture

**Fig. 2:**
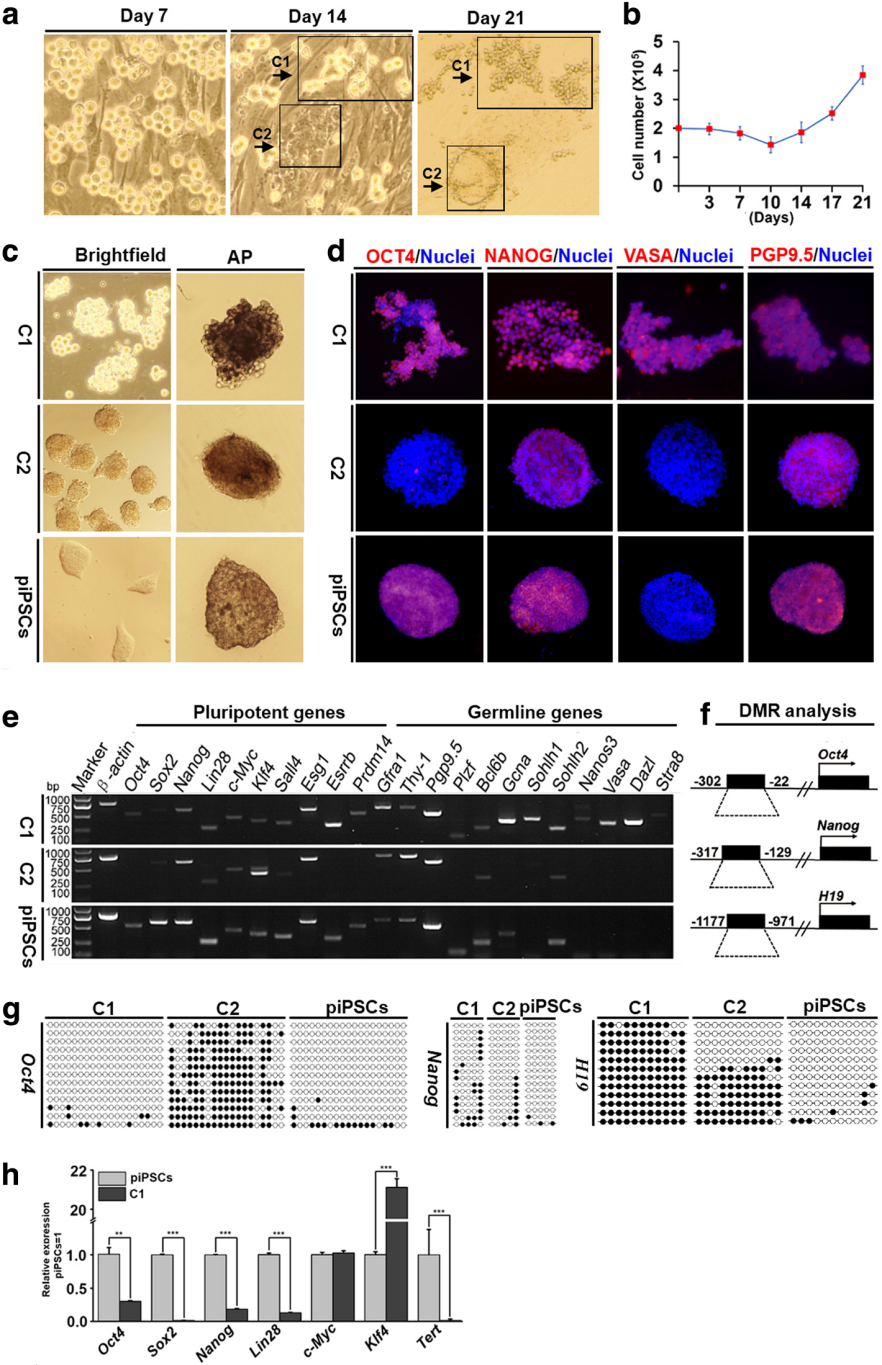
Culture and identification of purified undifferentiated putative porcine mGSCs. **a** The putative mGSCs were grown on mouse embryonic fibroblast feeder cells, and images were taken on days 7, 14, and 21 of culture. Two distinct stem cell-like clusters, termed as C1 and C2, appeared at day 14 of culture and became more obvious at day 21 of culture. **b** A graph shows the fluctuations in total cell numbers in the mGSC culture, revealing an increase in cell number toward the end of the culture period. **c** Clusters of C1 and C2 were separated and collected for AP activity analysis and compared with porcine-induced pluripotent stem cells (piPSCs) as a positive control. **d** Images of immunofluorescence analysis show expression of OCT4, NANOG, VASA, and PGP9.5 in the C1 and C2; nuclei were stained by Hoechst 33,342 (blue). **e** RT-PCR was performed to analyze the expression of pluripotent and germline genes in the C1 and C2. **f** A schematic indicates the differentially methylated regions (DMRs) of *Oct4*, *Nanog*, and *H19* used for analysis in the C1 and C2. **g** A diagram shows the bisulfite sequencing results for each gene. Each line represents an individual clone sequencing result, and each circle represents one CpG site. The solid circles indicate a methylated CpG, whereas an empty circle indicates an unmethylated CpG. **h** Quantitative PCR was performed to show the relative mRNA expression levels of reprogramming factors (*Oct4*, *Sox2*, *Nanog*, *Lin28*, *c-Myc*, *Klf4*, and *Tert***)** between the C1 and piPSCs (*n* = 3). The data are expressed as the mean ± SEM. *P < 0.05

After 7 days of culture, the isolated putative porcine mGSCs congregated together on the mouse embryonic fibroblast feeder cells (Fig. 2a). Only a small portion of the putative porcine mGSCs was proliferative (Additional file 4: Figures S2b and Additional file 5: Figure S3). Surprisingly, at day 14 of culture, we observed two types of stem cell clusters with distinct morphologies (Fig. 2a), labeled as C1 and C2 here. The C1 had a similar morphology to that of the previously described mouse mGSCs, showing a grape-like appearance; however, the C2 exhibited a three-dimensional morphology similar to those of human testis-derived mesenchymal stem cells and the previously described porcine mGSCs (Fig. 2a). By day 21 of culture, both the C1 and C2 grew into larger clusters (Fig. 2a), and the cell numbers increased (Fig. 2b).

To further characterize the C1 and C2, and determine which might be the putative mGSCs, the cell clusters were analyzed by AP staining and expression of specific pluripotency-associated mGSC markers. The piPSCs were used as a positive control. The C1 and C2 exhibited strong AP staining, similar to piPSCs (Fig. 2c). Of critical importance, the C1, but not the C2, expressed the pluripotent factor OCT4 and the germline-specific marker VASA (Fig. 2d). Both the C1 and C2 expressed NANOG and PGP9.5 as observed in piPSCs. Consistently, other pluripotency-associated markers, including SSEA1, SSEA4, TRA-1-60, and TRA-1-81, were expressed both in the C1 and C2 and the piPSCs, whereas only the C1 expressed GFRA1 and PLZF (Additional file 6: Figure S4).

We found that the C1 and C2 displayed distinct patterns of gene expression by RT-PCR (Fig. 2e). The C1 expressed the pluripotent factors *Oct4*, *Sox2*, *Nanog, Lin28*, *c-Myc*, *Klf4*, *Sall4*, *Esg1*, *Esrrb*, and *Prdm14* at similar levels to those observed in piPSCs, but they also expressed germ cell-specific factors, such as *Plzf*, *Bcl6b*, *Gcna*, *Sohlh1*, *Sohlh2*, *Nanos3*, *Vasa*, *Dazl*, and *Stra8*. The C2, however, did not express important pluripotent factors, such as *Oct4*, *Esrrb*, and *Prdm14*, and also failed to express many germ cell-specific markers, including *Plzf*, *Gcna*, *Sohlh1*, *Nanos3, Vasa*, *Dazl*, and *Stra8*.

By performing bisulfite sequencing analyses (Fig. 2f), the C1 showed low-level (4.04%) methylation of the *Oct4* promoter and relatively high-level (up to 30%) methylation of the *Nanog* promoter (Fig. 2g). Importantly, *H19*, which is normally methylated at the paternal allele, was completely methylated in the C1. Conversely, the C2 exhibited higher methylation levels (68.82%) of the *Oct4* promoter consistent with the absence of *Oct4* transcripts in the C2 by RT-PCR (Fig. 2e); there was also lower methylation (12.50%) of the *Nanog* promoter, compared with the C1 (Fig. 2g). The C2 displayed 50.75% methylation of the *H19* promoter, which is the expected epigenetic status in somatic cells. The positive control piPSCs showed very low methylation levels at the *Oct4*, *Nanog*, and *H19* promoters (Fig. 2g).

We further compared the mRNA expression of reprogramming factors for piPSC generation between piPSCs and the C1 by quantitative RT-PCR (Fig. 2h). With the exception of *Klf4* and *c-Myc*, the expression of *Oct4*, *Sox2*, *Nanog*, *Lin28*, and *Tert* in the C1 was significantly lower than the piPSCs. After differentiation induction, the piPSCs, but not C1, generated embryoid bodies and expressed the multilineage-associated genes *Sox3*, *Scta2*, and *Afp* of endoderm, mesoderm, and ectoderm, respectively (Additional file 7: Figure S5).

### The RNA-seq analysis of C1 and C2

**Fig. 3:**
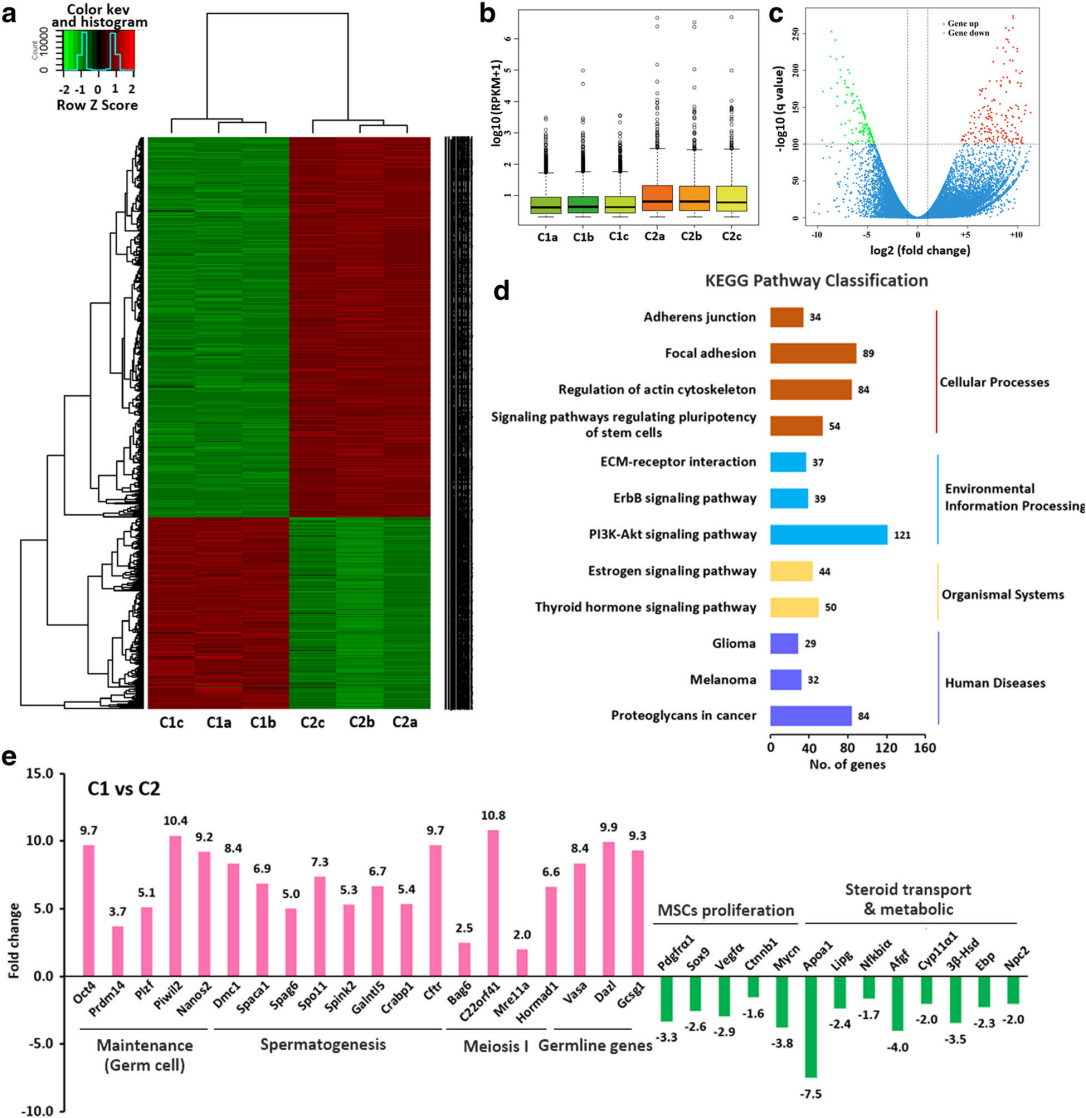
The RNA-seq analysis showed distinct difference between C1 and C2. **a** Heat map; **b** Boxplot; **c** Volcano plot; **d** KEGG pathway enrichment showed that they varied in cellular processes, environmental information, organismal system pathways; **e** Among the differentially-expressed genes, C1 showed higher expression of genes involved in the maintenance of germ cells, spermatogenesis, and meiosis, and regulation of germline stem cells than did C2, whereas genes involved in mesenchymal stem cells proliferation and steroid transport and metabolism had higher expression in C2 than in C1

There were distinct differences in gene expression between C1 and C2, with a total of 4771 DE genes (Fig. 3a, c), which indicated the different origins of C1 and C2 (Fig. 3b). The KEGG pathway enrichment showed that these DE genes were enriched in pathways of cellular processes, environmental information, organismal system, adherens junction, focal adhesion, regulation of actin cytoskeleton, signaling pathways regulating pluripotency of stem cells, ECM-receptor interaction, ErbB signaling pathway, PI3K-Akt signaling pathway, estrogen signaling pathway, thyroid hormone signaling pathway, glioma, melanoma and proteoglycans in cancer (Fig. 3d and Additional file 8: Figure S6). Among the DE genes, C1 had higher expression of genes involved in maintenance of germ cells, spermatogenesis, and meiosis, and regulation of germline stem cell than did C2, whereas genes involved in mesenchymal stem cells proliferation and steroid transport and metabolism had higher expression in C2 than in C1 (Fig. 3e).

### Induction of sperm differentiation in the C1 and C2 clusters

**Fig. 4:**
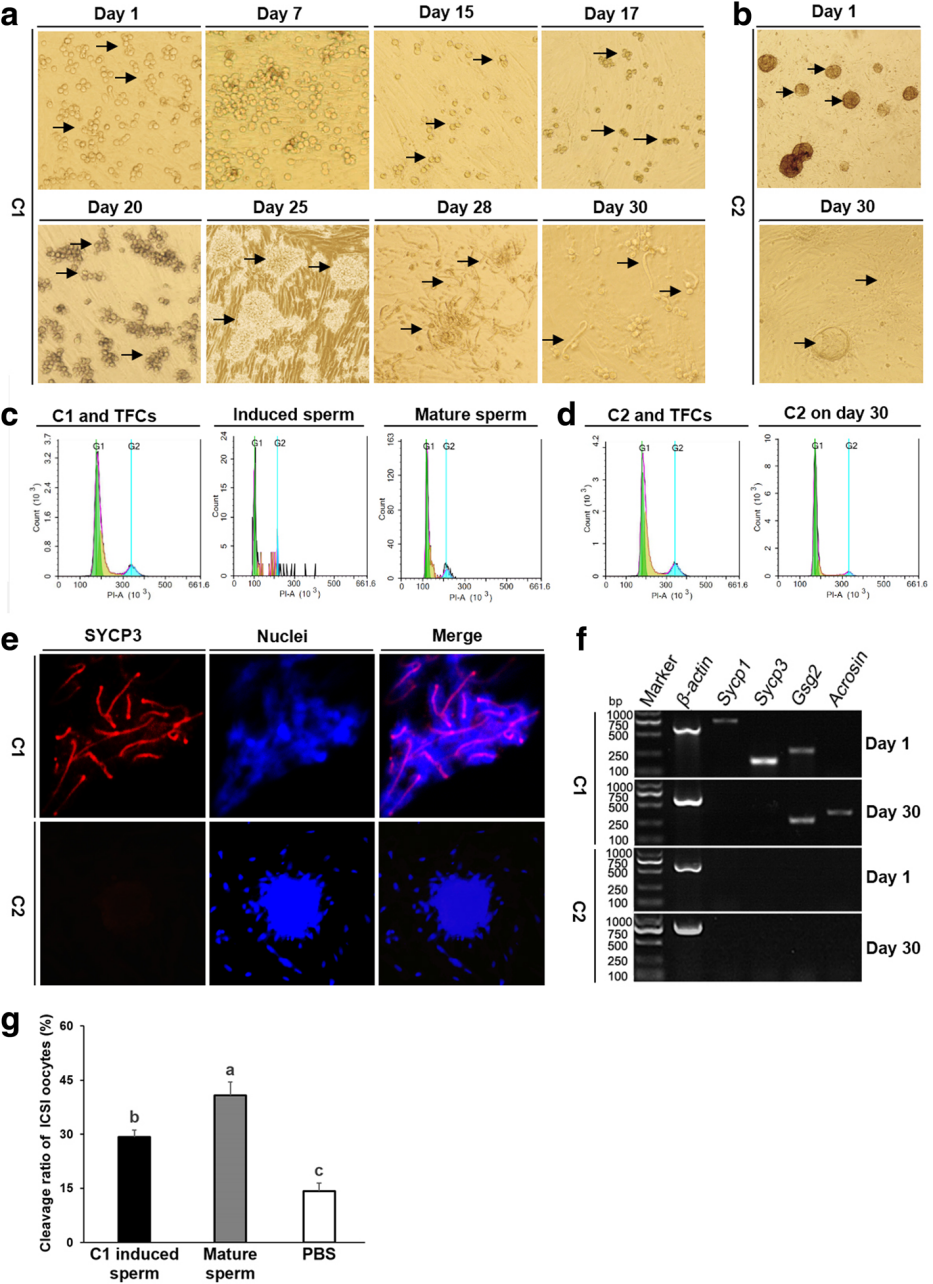
Induction of sperm differentiation in C1 and C2 clusters. **a** The C1 were cultured on feeder cells, and they formed A_pr_ spermatogonia (2-cell), A_al-4_ spermatogonia (4-cell), and A_al-8_ and A_al-16_ spermatogonia (8-cell and 16-cell) at days 15, 17, and 20 of culture, respectively. The majority of differentiated spermatogonia were observed at days 25–28 of culture, whereas the sperm cells with long tails were largely observed at day 30 of culture. **b** Images show the sperm differentiation induction of the C2 at days 1 and 30. **c** Ploidy analyses were performed by flow cytometry using the C1 and testicular fibroblast cells, induced sperm harvested at day 30 of culture, and live sperm harvested from a healthy boar as positive control. **d** Ploidy analyses were performed by flow cytometry of the C2 at days 1 and 30, and the testicular fibroblasts. **e** Expression of the meiosis-associated marker SYCP3 was detected in the induced C1, but not the induced C2. The nuclei were stained with Hoechst 33,342 (blue). **f** RT-PCR analysis shows expression of sperm-specific markers in the C1 and C2 at the beginning (day 1) and end (day 30) of sperm differentiation induction. **g** The induced sperm can activate the oocytes to initiate early embryonic development after intracytoplasmic sperm injection (ICSI), and exhibited significant differences compared to the negative group (PBS) and the positive group (mature sperm). The ability of the induced sperm to activate the oocytes was lower than that of mature sperm, with lower efficiency of blastocyst production

To identify the origins of the C1 and C2, we further investigated their capacity to differentiate into spermatozoa in vitro. The C1 clusters began to assemble together to differentiate on day 7 of culture (Fig. 4a). They had divided into paired (A_pr_) spermatogonia (2 couplets) on day 15, and had formed aligned (A_al-4_) spermatogonia (4 couplets) by day 17. The A_al-8_ and A_al-16_ spermatogonia (8 and 16 couplets) observed on day 20, maintained rapid division on day 25. At days 28 and 30, an increasing number of sperm-like cells with long tails were apparent. In contrast, we observed no evidence of sperm differentiation from the C2 during days 1 to 30 of culture under the same induction conditions (Fig. 4b). Ploidy analyses showed that the induced sperm cells were monoploid (first cycle at 100, n referring to DNA content) and appeared similar to a boar semen control, whereas the C1 clusters and testicular fibroblast cells were diploid (first cycle at 200, 2n referring to DNA content), as expected (Fig. 4c). The C2, before and after treatment to induce differentiation, were unequivocally diploid (Fig. 4d), consistent with the C2 clusters failing to undergo sperm differentiation (Fig. 4b).

Furthermore, the induced C1 clusters showed strong staining of a marker for spermatocyte, meiosis protein synaptonemal complex protein 3 (SYCP3) (Fig. 4e). The induced C2 clusters, however, showed no evidence of SYCP3, and hence failure to undergo meiosis (Fig. 4e). Furthermore, the C1, but not the C2, expressed the sperm-specific markers *Acrosin* and *Gsg2* after induction (Fig. 4f). Finally, the induced sperm from C1 clusters were injected into matured porcine oocytes by intracytoplasmic sperm injection (ICSI), with the normal mature sperm as the positive control and PBS as negative control (Fig. 4g). The oocytes were all activated in these three groups, and the cleavage ratio of ICSI oocytes by the induced sperm from C1 clusters, while lower than the positive control, was nonetheless higher than the negative control (Fig. 4g). Collectively, these results indicate that the C1 clusters, similar to the mouse mGSC-like clusters, are indeed the porcine mGSCs.

### Identification of the C2 clusters as porcine PLCs

**Fig. 5:**
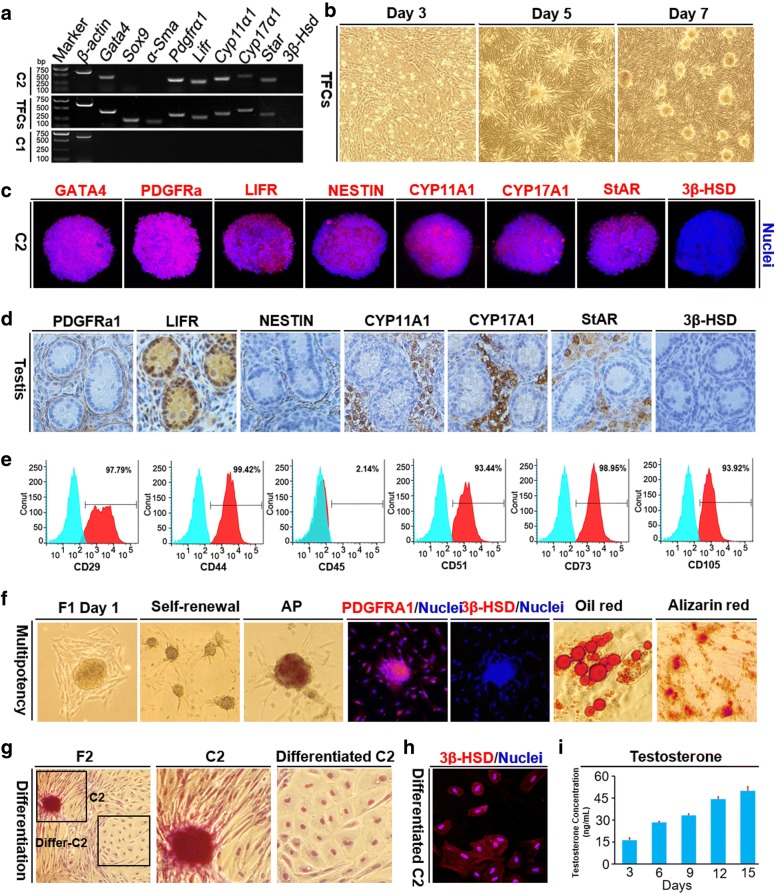
Identification of the C2 clusters as progenitor Leydig cells (PLCs) derived from the porcine testis. **a** RT-PCR analysis showed that the C2 expressed Leydig cells (LC)-specific genes, such as *Gata4*, *Pdgfrα1*, *Lifr*, *Cyp11α1*, *Cyp17α1*, and *Star*, but did not express the Sertoli cell (SC)-specific marker *Sox9* or the peritubular myoid cell (PTM) marker *α-Sma*. **b** Images show that, by day 7 of culture without germ cells, the testicular fibroblasts formed clusters that appeared similar to the C2. **c** Immunofluorescent staining showed that GATA4, PDGFRA1, LIFR, NESTIN, CYP11A1, CYP17A1, and StAR, were all expressed in the C2. The nuclei were stained with Hoechst 33,342 (blue). **d** Immunohistochemistry staining showed the expression levels of PDGFRA1, LIFR, NESTIN, CYP11A1, CYP17A1, StAR, and 3β-HSD in porcine testicular tissues from 5-day-old piglets. **e** Flow cytometric analyses showed the expression of mesenchymal stem cell-specific markers (CD29, CD44, CD51, CD73, and CD105) but no expression of hematopoietic marker CD45 in the C2. **f** Multipotency analyses showed that the C2 maintained multipotency after several passages and displayed strong AP activity. Immunofluorescent staining showed that the C2 expressed PDGFRA1 (Red), but not 3β-HSD. The nuclei were stained with Hoechst 33,342 (blue). Oil red and Alizarin red staining showed they had the capacity to differentiate into adipogenic and osteogenic lineages, respectively. **g** Images of the C2 following adult Leydig cell (ALC) differentiation induction. **h** Immunofluorescent staining showed 3β-HSD expression in the induced ALCs. **i** The testosterone concentration was increased during the induction of ALC differentiation (*n* = 6). The data are expressed as mean ± SEM

We next analyzed the possible origin of the C2 by comparing the level of expression of specific markers for Sertoli cells, peritubular myoid cells and Leydig cells to those in the C1. Testicular fibroblasts, which express all of the analyzed genes except *3β-Hsd*, were used as positive control. Interestingly, the C2 expressed Leydig cells-specific markers, including *Gata4*, *Pdgfrα1*, *Lifr*, *Cyp11α1*, *Cyp17α1*, and *Star*, but did not express the Sertoli cells marker *Sox9* or the peritubular myoid cells marker *α-Sma*, whereas the C1 did not express these genes (Fig. 5a).

We further observed that testicular fibroblasts cultured without germ cells for 7 days formed clusters that were similar to the previously observed C2 (Fig. 5b). These cells were highly proliferative, and showed a low rate of apoptosis (Additional file 9: Figure S7), indicating that the C2 may be derived from testicular fibroblasts. Furthermore, the C2 expressed all of the Leydig cells-specific markers analyzed, including GATA4, PDGFRA1, LIFR, NESTIN, CYP11A1, CYP17A1, and StAR, except for 3β-HSD (Fig. 5c). The PDGFRA1, LIFR, and NESTIN proteins were expressed adjacent to the seminiferous tubules, whereas the markers of more differentiated Leydig cells, or PLCs, including CYP11A1, CYP17A1, and StAR, were expressed in the interstitial space of the porcine testis (Fig. 5d). There was no expression of 3β-HSD in the C2 or in the porcine testis (Fig. 5c, d).

Moreover, the passaged C2 also expressed markers of mesenchymal stem cells, including CD29, CD44, CD51, CD73, and CD105, and were negative for the hematopoietic marker CD45 (Fig. 5e). The C2 were passaged to a new culture dish without feeder cells and displayed similar morphology as fibroblast-like cells (Fig. 5f). The three-dimensional morphology initially disappeared, but was subsequently regained, and the C2 with three-dimensional morphology remained in an undifferentiated state. These C2 clusters displayed AP activity, and exhibited the capacity to undergo adipogenic and osteogenic differentiation, as revealed by strong staining with Oil red and Alizarin red, respectively (Fig. 5f).

The passaged C2 expressed the Leydig cell marker PDGFRA1, but not a marker of more differentiated Leydig cells, 3β-HSD (Fig. 5f). However, the C2 were efficiently induced to differentiate into adult Leydig cells with the addition of LH and cholesterol, which are necessary for testosterone biosynthesis (Additional file 10: Figure S8). Beginning at day 12 of differentiation induction, the amounts of round adult Leydig cells were increasing (Additional file 10: Figure S8e), which became more abundant by days 15 to 20 after induction (Fig. 5g and Additional file 10: Figure S8f). The induction of adult Leydig cells was confirmed by the presence of differentiated Leydig cells markers (Fig. 5h and Additional file 11: Figure S9), including 3β-HSD, CYP11A1, CYP17A1, StAR, and the loss of the PLC markers PDGFRA1 and LIFR. Moreover, testosterone concentrations increased progressively throughout the differentiation induction of C2 into adult Leydig cells, proving their successful differentiation (Fig. 5i). Collectively, these results provide comprehensive evidence that the C2 are indeed PLCs.

## Discussion

The testis contains several functionally important, organ-specific cell types, including germ cells and Sertoli cells within the seminiferous tubules, Leydig cells within the interstitial compartment, and peritubular myoid cells that surround the seminiferous tubules. Among them, mGSCs, which are maintained at a limited number in the mammalian testis by balancing self-renewal and spermatogenesis [22], have attracted increasing attention due to their great promise for use in reproductive medicine. It is well recognized that ES-like cells can be generated from mouse mGSCs [23–25], but whether they can be generated from the mGSCs of human or other species remains controversial.

The first step in the enrichment of mGSCs, including gonocytes and spermatogonia stem cells, is to separate testicular cells. Previous investigations have shown that mGSCs can be successfully isolated from mice [26], rats [27], pigs [28], and humans [29]. Cultured mouse mGSCs, however, exhibit different characteristics to those of human [30, 31] and domestic animals, including pig [11, 32, 33], goat [12, 34, 35], cattle [13, 36], and chicken [37]. The reason why mGSCs displayed distinct morphological differences among species remains unclear. Reliable molecular markers to identify and enrich for mGSCs are crucial for the successful establishment of mGSC in vitro culture, because nonspecific markers may lead to the enrichment and cultivation of other cell types that also express them. For example, the human putative mGSC clusters with three-dimensional morphology, which were harvested using GFRA1, CD49f, and SSEA1 as enrichment markers, were subsequently demonstrated to be testis-derived mesenchymal stem cells [9, 38, 39]. The ES-like cells derived from human adult putative mGSCs [38–40] were later proven to be testis-derived mesenchymal stem cells [6, 7, 9, 41], questions regarding the true identity of these cells have remained for years. Primate testis-derived mesenchymal stem cells express many markers that were previously considered to be mGSC-specific, such as GFRA1, GPR125, THY-1 (CD90), ITGA6, SSEA4 TRA-1-81 and PGP9.5, further suggesting that these markers are not suitable for the isolation and identification of mGSCs [6].

It remains unclear which markers will be effective for the isolation and enrichment of porcine mGSCs. The culturing porcine gonocytes isolated using SSEA1 as a marker [32], displayed morphological characteristics similar to those of human testis-derived mesenchymal stem cells, suggesting they were not mGSCs [11, 32] but Leydig cells [42]. For undifferentiated spermatogonia, PGP9.5 [43] and PLZF [44] have been claimed to be specific markers. However, PGP9.5, previously used to detect undifferentiated mGSCs in mouse [45], cow [46], sheep [47], and pig [43], has been shown to also be expressed in testis-derived mesenchymal stem cells [6]. Our isolated putative mGSCs displayed AP activity and expressed self-renewal markers, such as GFRA1, OCT4, and PLZF, which are reliable markers of undifferentiated porcine mGSCs. They also expressed critical pluripotent markers of pluripotent stem cells, such as NANOG, SSEA1, SSEA4, TRA-1-60, and TRA-1-81, and characteristic mGSC markers, such as VASA, PGP9.5, and CD29, similar to those of mouse mGSCs.

Here, we identified two different stem cell clusters, C1 and C2, upon culturing porcine testicular cells. These stem cell clusters shared some common, but many unique markers. We showed that PGP9.5, SSEA1, SSEA4, TRA-1-60, and TRA-1-81 were expressed in C1 as well as in C2 and piPSCs, indicating they are not specific markers for porcine mGSCs. The most distinct markers for identifying germ cells are the evolutionarily conserved germ cell-specific genes that are all important for spermatogenesis, including *Gcna*, *Sohlh1*, *Nanos3*, *Vasa*, *Dazl*, and *Stra8*. Expression of these germ cell-specific genes are landmarks for the identification of mGSCs. Similar to mouse mGSCs, the C1 expressed both pluripotency-associated and germ cell-specific genes, and exhibited AP activity. In contrast, the C2 did not express the crucial pluripotent factors *Oct4*, *Esrrb*, and *Prdm14* or the germ cell-specific genes, but they did display AP activity. The RNA-seq analyses also demonstrated distinct difference of gene expression between C1 and C2. The C1 had higher expression of genes involved in maintenance of germ cells, spermatogenesis, and meiosis, and regulation of germline stem cell, whereas C2 had higher expression of genes involved in mesenchymal stem cells proliferation and steroid transport and metabolism. Furthermore, the paternally imprinted gene *H19* was completely methylated in the C1, further indicating that the C1 possessed the characteristics of mGSCs. Most functionally discriminating C1, but not C2, showed the capacity to undergo differentiation to spermatozoa and were able to activate oocytes to develop into early embryos. Collectively, we have identified a method to isolate and cultivate, for the first time, porcine mGSCs (C1) that actually possess characteristics similar to those of mouse mGSCs [48], but quite different from those of the previously described porcine mGSCs [49].

During porcine testicular cell culture, we have also identified three-dimensional colonies of C2, morphology of which resembled the previously claimed porcine mGSCs, and found that the C2 expressed several markers of mGSCs and ES-like cells. Several groups have reported the successful derivation of ES-like cells from the adult human testis [38–40], but subsequent studies indicated that the clusters of haGSCs are not pluripotent stem cells [8, 9] and were later demonstrated to be testis-derived mesenchymal stem cells [41]. Other studies have shown that mGSC-derived clusters with similar morphologies to those of ES-like clusters can be easily covered up by the overwhelming number of surrounding somatic cells [50]. Therefore, we cultured the testicular fibroblasts without mGSCs for 7 days and observed the formation of a large number of three-dimensional clusters, which appeared to be similar to the C2. These data suggest that the C2 may be of mesenchymal origin. Upon passaging to the next generation without feeder cells, the cells of the C2 appeared to be more similar to fibroblasts and began to rapidly proliferate, much faster than mGSCs. Indeed, culturing mixtures of cell types may result in dilution of the slowly-growing mGSCs, particularly if the other cells are rapidly proliferating, such as testis-derived mesenchymal stem cells.

Leydig cells are the main testosterone-producing cells residing in the interstitium of the testis and are vital for the development of spermatogenesis and the male phenotype [51]. Stem Leydig cells were characterized based on their capability of self-renewal and to differentiate into PLCs [52]. The spindle-shaped PLCs originating from stem Leydig cells do not express 3β-HSD, they have proliferative ability, and show morphologies and express stem cell markers similar to those of stem Leydig cells, but ultimately, they differentiate into immature Leydig cells and then adult Leydig cells [53]. Unlike stem Leydig cells, however, PLCs express enzymes related to steroid metabolism including CYP11A1, CYP17A1, and StAR, and modestly express the luteinizing hormone receptor (LHR). The expression of 3β-HSD in immature and mature Leydig cells suggests the synthesis and secretion of large quantities of testosterone [54]. Rat stem Leydig cells were distinguished by their negative expression of 3β-HSD and LHR, and later adult Leydig cells from the rat testis were also identified [51, 55], but very little is known regarding the identification of porcine PLCs.

To identify the origin of the C2, we showed that the C2 expressed specific markers of Leydig cells and stem Leydig cells (PDGFRA1 and LIFR), and expressed steroid metabolism-related genes (CYP11A1, CYP17A1, and StAR). Leydig cells in pigs within 2.5 weeks after birth show features of steroidogenic capacity, such as high 3β-HSD expression [56]. We found that the C2 did not express 3β-HSD. Further analyses showed that the C2 expressed specific markers of mesenchymal stem cells, CD29, CD44, CD51, CD73, and CD105. Our results are in accordance with previous studies with human mGSCs, which demonstrated that the human testis-derived stem cells were of mesenchymal origin [7]. Under our culture system, the C2 maintained self-renewal, grew into clusters, and were able to be differentiated into adipocytes and osteoblasts. The addition of LH differentiated C2 into adult Leydig cells that expressed 3β-HSD and secreted testosterone. These results provide evidence that the C2 are not ES-like cells or mGSCs, and do not correspond to the previously reported human mGSCs [8] but like mouse stem Leydig cells [57]. Collectively, we showed that even though the C2 showed morphologies similar to those of the previously described porcine mGSCs, they were in fact PLCs.

## Conclusion

We have identified two distinct testis-derived stem cell populations, mGSCs and PLCs, during porcine testicular cell culture. We found that the porcine mGSCs and PLCs were not pluripotent, and they displayed an overlap of markers, which may be the cause of misleading results of in vitro testis-derived stem cell studies in the past. We demonstrated, for the first time, that the previously claimed porcine mGSCs were in fact PLCs, and the actual porcine mGSCs, obtained here, showed characteristics similar to those of mouse mGSCs. Both mGSCs and PLCs are very important for regulating the male reproductive development. These results may provide new insights into the previous misleading recognition of porcine mGSCs, and establish identifications of porcine testis-derived stem cells, as well as help facilitate the application of testis-derived stem cells in human reproductive medicine and animal breeding. Accurately identified testis-derived stem cell populations will aid in avoiding misinterpretation of data, crucial for future application of stem cells in reproductive medicine.

## Additional files

Additional file 1: **Table S1.** Primary and secondary antibodies used for immunofluorescence or FACS analysis. (DOCX 15 kb)

Additional file 2: **Table S2.** Primer sequence, target product size, and accession number of target genes. (DOCX 22 kb)

Additional file 3: **Figure S1.** Enrichment of porcine male germline stem cell (mGSCs). (**a**) The testicular suspension contained several cell types. (**b**) Testicular fibroblast cells (TFCs) were removed by differential attachment technique. (**c**) The unattached cells underwent density gradient centrifuging to remove the leukomonocytes (LYMs) and cell debris (**d**) and red blood cells (RBCs) (**e**), and to retain mGSCs (**f**). (JPG 699 kb)

Additional file 4: **Figure S2.** Immunolocalization of VASA and NANOG proteins in the testis of 5-day-old piglet. Immunohistochemistry analysis showed that the reproduction-associated marker VASA localized to the basement membrane of seminiferous tubules, whereas the pluripotent factor NANOG was expressed in the interstitial space and seminiferous tubules. (JPG 442 kb)

Additional file 5: **Figure S3.** Cell cycle assay of putative porcine male germline stem cell (mGSCs). Cell cycle analyses at cell culture days 3, 5, and 7 revealed that only a small percentage of the putative mGSCs entered the S and G2 cell division phases. Meanwhile, part of putative mGSCs underwent apoptosis detected by analysis of apoptosis at day 7 of cell culture. (JPG 285 kb)

Additional file 6: **Figure S4.** Expression of pluripotency-associated markers and germ cell-specific markers in the C1 and C2 clusters. Immunofluorescent staining showed that both the C1 and C2 clusters expressed the pluripotency-associated markers SSEA1, SSEA4, TRA-1-60, and TRA-1-81. However, the C1 clusters, but not the C2 clusters, expressed the germ cell-specific markers GFRA1 and PLZF. (JPG 249 kb)

Additional file 7: **Figure S5.** Comparison of pluripotency potential between the C1 clusters and porcine-induced pluripotent stem cells (piPSCs). (**a)** The C1 clusters and (**b**) piPSCs were cultured without feeder cells and serum for 7 days to induce embryoid body formation. Embryoid bodies were formed from the piPSCs, but not the C1 clusters, (**c**) with induction of lineage-specific genes. (JPG 310 kb)

Additional file 8: **Figure S6.** Gene ontology (GO) analysis of the differentially expressed genes between C1 and C2. (JPG 403 kb)

Additional file 9: **Figure S7.** Cell cycle assay of putative porcine progenitor Leydig cells (PLCs). Cell cycle analysis of the PLCs showed that they had rapidly dividing capacities at days 3, 5, and 7 in culture. Analysis of apoptosis showed that PLCs had very low levels of cell death at day 7 in culture (99.81% propidium iodide, annexin V double negative). (JPG 299 kb)

Additional file 10: **Figure S8.** Induction of C2 clusters differentiation into mature adult Leydig cells (ALCs). The C2 clusters began to differentiate into fibroblast cells at day 1 (**a**), became smaller from days 3 to 6 (**b**, **c**), and had disappeared by day 9 (**d**) The C2 clusters were fully differentiated into ALCs by day 12 (**e**), with abundant mature ALCs observed by day 15 (**f**). (JPG 558 kb)

Additional file 11: **Figure S9.** Expression of adult Leydig cell (ALC)-associated markers in induced differentiated C2 clusters. They did not express the PLC markers PDGFRA1 (**a**) and LIFR (**b**), but expressed testosterone synthesis enzymes CYP11A1 (**c**), CYP17A1 (**d**), and StAR (**e**), demonstrated at both the protein and mRNA levels (**f**). (JPG 301 kb)

## Abbreviations

3β-HSD: β hydroxysteroid dehydrogenase
AP: Alkaline phosphatase
bFGF: Basic fibroblast growth factor
BSA: Bovine serum albumin
c-KIT: Stem cell factor receptor
COCs: Cumulus-oocyte complexes
CYP11A1: Cytochrome P450 family 11 subfamily A member 1
CYP17A1: Cytochrome P450 family 17 subfamily A member 1
DE: Differentially expressed
DMEM: Dulbecco’s modified Eagle medium
DPBS: Dulbecco’s phosphate buffered saline
EGF: Epidermal growth factor
ES: Embryonic stem
FBS: Fetal bovine serum
GATA4: GATA binding protein 4
GDNF: Glial cell line-derived neurotrophic factor
GFRA1: Glial cell line-derived neurotrophic factor family receptor alpha-1
GO: Gene ontology
haGSCs: Human testis-derived ES-like cells
ICSI: Intracytoplasmic sperm injection
ITS: Insulin-transferrin-selenium
KEGG: Kyoto encyclopedia of genes and genome
LH: Luteinizing hormone
LHR: LH receptor
LIF: Leukemia inhibitory factor
LIFR: LIF receptor
LYMs: Leukomonocytes
mGSCs: Male germline stem cells
NBT/BCIP: Nitro blue tetrazolium and 5-bromo-4-chloro-3-indolyl-phosphate
NESTIN: NES gene
OCT4: Octamer-binding transcription factor 4
PBS: Phosphate buffer saline
PDGF-BB: Platelet-derived growth factor-BB
PDGFRA1: Platelet-derived growth factor receptor A
PI: Propidium iodide
piPSCs: Porcine-induced pluripotent stem cells
PLCs: Progenitor Leydig cells
PLZF: Promyelocytic leukemia zinc finger
PTM: peritubular myoid cell
PZM-3: Porcine zygote medium 3
RBCs: Red blood cells
RT-PCR: Reverse transcriptase
SSEA1/4: Stage-specific embryonic antigens 1/4
StAR: Steroidogenic acute regulatory protein
SYCP3: Synaptonemal complex protein 3
TFCs: Testicular fibroblast cells
VASA: DEAD-box polypeptide 4
β-ME: β-mercaptoethanol
