# Supplementary material for: Previously claimed male germline stem cells from porcine testis are actually progenitor Leydig cells
Source: Stem Cell Res Ther. 2018 Jul 18;9:200. doi: 10.1186/s13287-018-0931-0 (PMC6052628; doi:10.1186/s13287-018-0931-0)
Supplement: Supplementary file 1 — Table S1. Primary and secondary antibodies used for immunofluorescence or FACS analysis. (DOCX 15 kb) [file 13287_2018_931_MOESM1_ESM.docx]

**Additional file 1 Table S1.** Primary and secondary antibodies used for immunofluorescence or FACS analysis.

| Antibody | Dilution | Supplier |
| --- | --- | --- |
| Anti-GFRA1 (E-11) mouse IgG | 1: 200 | Santa Cruz, Heidelberg (sc-271546) |
| Anti-OCT4 mouse IgG | 1: 200 | Millipore, Germany (MAB360) |
| Anti-PLZF (D-9) mouse IgG | 1: 200 | Santa Cruz, Heidelberg (sc-28319) |
| Anti-c-KIT rat IgG | 1: 100 | Millipore, Germany (MAB4344) |
| Anti-NANOG rabbit IgG | 1: 200 | Cell Signaling, USA (D73G4) |
| Anti-PGP9.5 rabbit IgG | 1: 100 | Millipore, Germany (AB5925) |
| Anti-VASA mouse IgG | 1: 200 | Abcam, Cambridge (AB13840) |
| Anti-SYCP3 rabbit IgG | 1: 200 | Abcam, Cambridge (AB15093) |
| Anti-GATA4 mouse IgG | 1: 100 | Millipore, Germany (MAB4344) |
| Anti-PDGFRa1 rabbit IgG | 1: 100 | Abcam, Cambridge (AB61219) |
| Anti-LIFR rabbit IgG | 1: 200 | Bioss, USA (bs-1458R) |
| Anti-NESTIN rabbit IgG | 1: 100 | Millipore, Germany (MAB4344) |
| Anti-CYP11A1 rabbit IgG | 1: 200 | Liankebio, China (AB2060) |
| Anti-CYP17A1 rabbit IgG | 1: 200 | Liankebio, China (AB1766) |
| Anti-StAR rabbit IgG | 1: 200 | Cell Signaling, USA (D10H125) |
| Anti-3β-HSD rabbit IgG | 1: 100 | Abcam, Cambridge (AB61219) |
| Anti-SSEA1 mouse IgG | 1: 200 | Developmental Studies Hybridoma  Bank, Iowa (MC-480) |
| Anti-SSEA4 mouse IgG | 1: 200 | Developmental Studies Hybridoma  Bank, Iowa (MC-813-70) |
| Anti-TRA-1-60 mouse IgM | 1: 100 | Millipore, Germany (MAB4360) |
| Anti-TRA-1-81 mouse IgM | 1: 100 | Millipore, Germany (MAB4381) |
| Anti-CD29 mouse IgG | 1: 200 | BD Pharmingen, USA (552369) |
| Anti-CD44 rat IgG | 1: 200 | Abcam, Cambridge (ab119863) |
| Anti-CD45 (3H1362) mouse IgG | 1: 100 | Santa Cruz, Heidelberg (sc-70690) |
| Anti-CD51 mouse IgG | 1: 200 | Millipore, Germany (MAB1976H ) |
| Anti-CD73 rabbit IgG | 1: 200 | Abcam, Cambridge (ab175396) |
| Anti-CD105 (3H1805) mouse IgG | 1: 100 | Santa Cruz, Heidelberg (sc-71043) |
| Goat Anti-rat IgG Alexa 488 | 1 :500 | Invitrogen (A11006) |
| Goat Anti-mouse IgG Alexa 488 | 1: 500 | Invitrogen (A11001) |
| Donkey Anti-mouse IgG Alexa 568 | 1: 500 | Invitrogen (A10037) |
| Goat Anti-rabbit IgG Alexa 488 | 1: 500 | Invitrogen (A11034) |
| Goat Anti-rabbit IgG Alexa 568 | 1: 500 | Invitrogen (A11036) |
| Goat anti-Mouse IgM Alexa 488 | 1: 500 | Invitrogen (A-21042) |
| Goat anti-Mouse IgM Alexa 568 | 1: 500 | Invitrogen (A-21043) |
