# Supplementary material for: Previously claimed male germline stem cells from porcine testis are actually progenitor Leydig cells
Source: Stem Cell Res Ther. 2018 Jul 18;9:200. doi: 10.1186/s13287-018-0931-0 (PMC6052628; doi:10.1186/s13287-018-0931-0)
Supplement: Supplementary file 2 — Table S2. Primer sequence, target product size, and accession number of target genes. (DOCX 22 kb) [file 13287_2018_931_MOESM2_ESM.docx]

**Additional file 2 Table S2.** Primer sequence, target product size, and accession number of target genes.

| Gene | Primer sequence (5’-3’) | | | | Product size (bp) | Accession No. | |
| --- | --- | --- | --- | --- | --- | --- | --- |
| *β-actin* | F: | CCGTGAGAAGATGACCCAGATCATG | | | 612 | AY550069 | |
|  | R: | CGTGATCTCCTTCTGCATCCTGTC | | |  |  |  |
| *Oct4* | F: | GGGGCTCACTTTGGGGGTTCTCT3 | | | 521 | NM_001113060 | |
|  | R: | CAGGGAATGGGACCGAGGAGTACA | | |  |  |  |
| *Sox2* | F: | CGGCGGTGGCAACTCTACTG | | | 604 | NM_001123197 | |
|  | R: | GGGCGAGCCGTTCATGTAGGT | | |  |  |  |
| *Nanog* | F: | CTTATTCAGGACAGCCCTGATTCTTC | | | 614 | NM_001129971 | |
|  | R: | AAGACGGCCTCCAAATCACTG | | |  |  |  |
| *Lin28* | F: | GTTCGGCTTCCTGTCCAT | | | 269 | NM_001123133 | |
|  | R: | AGTTGTAGCACCTGTCTCCCT | | |  |  |  |
| *c-Myc* | F: | GCGGGCACGGCGGCTACT | | | 443 | NM_001005154 | |
|  | R: | GGGGGGCGCTGCATAATTGT | | |  |  |  |
| *Klf4* | F: | CGCGCATGTGCCCCAAGATC | | | 380 | NM_001031782 | |
|  | R: | CCGGGGCCACGACCTTCTC | | |  |  |  |
| *Sall4* | F: | GCCCACCACCTACGTCAA | | | 343 | NM_001114673 | |
|  | R: | TCCGTCCGTCCCTAACAG | | |  |  |  |
| *Esg1* | F: | AGAAAGGTGGACGATAAGGA | | | 628 | ENSSSCG00000005291 | |
|  | R: | GGTTTGGAGGAATTGTAGGT | | |  |  |  |
| *Esrrb* | F: | ATGAAATGCCTCAAAGTGGG | | | 315 | XM_001928051 | |
|  | R: | GGAGAAGCCTGGGATGTGC | | |  |  |  |
| *Prdm14* | F: | CGCCCCCAGTGGATGCTTCTCT | | | 541 | XM_003125600 | |
|  | R: | CGGGCACAGTTGACATAGGACATC | | |  |  |  |
| *Gfra1* | F: | GCCCTCAAACAGAAGTCG | | | 660 | ENSSSCG00000010655 | |
|  | R: | TGTCCCAATAAGCCCAGA | | |  |  |  |
| *Thy-1* | F: | CTTATCAGGCTTCACTACCAA | | | 648 | ENSSSCG00000015122 | |
|  | R: | CCTGCTGAAAGAGGTGCT | | |  |  |  |
| *Pgp9.5* | F: | ACAGAGAAGTTGTCTCCGGAAGA | | | 529 | AY459532 | |
|  | R: | GGAACGCTTTGCCATCAGA | | |  |  |  |
| *Plzf* | F: | AGGCTCGGTATCTCAAGAACATC | | | 144 | ENSSSCG00000030095 | |
|  | R: | TCATGGCTGAAAGACCAAACG | | |  |  |  |
| *Bcl6b* | F: | CGCTCCTCTTCTGGACTTCATG | | | 253 | XM_003131927 | |
|  | R: | CTCAGTAGGTGGGTCTGGGTGT | | |  |  |  |
| *Gcna* | F: | AGAAAGAGTCAGAGCCACCAAC | | | 372 | ENSSSCP00000008171 | |
|  | R: | CCATAGGAAAGCCATCATAGAG | | |  |  |  |
| *Sohlh1* | F: | CTTGGTGCTGGGGTGTGC | | | 427 | XM_003122284 | |
|  | R: | GCATGGTCACGCTGGCTATT | | |  |  |  |
| *Sohlh2* | F: | ATCGGTGGGCATGAAAGG | | | 206 | XM_001926611 | |
|  | R: | TCGGCAGTTGGTAAAGCAGT | | |  |  |  |
| *Nanos3* | F: | ACCAGTGCCAGGACCAGA | | | 421 | ENSSSCG00000013759 | |
|  | R: | ACAGCAGGGAGACGGAGA | | |  |  |  |
| *Vasa* | F: | GCCCAGGAATGCCATCAAAGGAA | | | 342 | AY626785 | |
|  | R: | CCCGCTCTCTCTGTTCCCGATCA | | |  |  |  |
| *Dazl* | F: | CCTCCTTACCCAAGTTCACC | | | 259 | XM_003358321 | |
|  | R: | CTGTCTGTATGCTTCGGTCC | | |  |  |  |
| *Stra8* | F: | CTACAAGCAGACGATGGACC | | | 489 | XM_003134656 | |
|  | R: | ATCTCATCATCAACGGGAAG | | |  |  |  |
| *Sycp1* | F: | TAAATGTAAATTGGACAAGAGTG | | | 803 | XM_003125829 | |
|  | R: | CCTTTGGTAATGGTGTAGATAA | | |  |  |  |
| *Sycp3* | F: | GGAGGATGTCATTGAAGGGAAG | | | 190 | ENSSSCG00000000863 | |
|  | R: | GGTTTTGAGAGAAGCCTTGGTGTA | | |  |  |  |
| *Acrosin* | F: | TTGTGGTCGTGGGCATCAC | | | 381 | NM_214033 | |
|  | R: | GGAAAGTGCTTGGGGAGGTT | | |  |  |  |
| *Gsg2* | F: | TGGGGCAGATGGGAAGAGTA | | | 246 | ENSSSCG00000026658 | |
|  | R: | TGGTGGTGATGAAGGAGGGA | | |  |  |  |
| *Gata4* | F: | CGCCGCCCGACACCCTAATC | | | 415 | AY115491 | |
|  | R: | GCCCGAGGGACCTGCTGACG | | |  |  |  |
| *Sox9* | F: | CCGCGAAGCCGTCAGCCAG | | | 240 | NM_213843 | |
|  | R: | GGCCGCTTCTCGCTCTCATTCAG | | |  |  |  |
| *α-Sma* | F: | AATGGCTCTGGGCTCTGT | | | 200 | DQ400922 | |
|  | R: | TTGGTGATGATGCCGTGT | | |  |  |  |
| *Pdgfrα1* | *F:* | GACCACCACGGCTCTAATG | | | 348 | XM_005674641 | |
|  | *R:* | CACAATCACCAGCAACACC | | |  |  |  |
| *Lifr* | F: | ACGAGCCTATACAGACGGAGG | | | 289 | XM_005672426 | |
|  | R: | ATTATTTGGGGTACAAGGATTCAT | | |  |  |  |
| *Cyp11α1* | F: | GCATCAAGCAGCAGGGTTC | | | 368 | NM_214427 | |
|  | R: | ATTTCCCAGGAGGCGGTAG | | |  |  |  |
| *Cyp17α1* | F: | GCTGGCACTGAGCACCTTT | | | 442 | NM_214428 | |
|  | *α*R: | TGCGTTCGTCTTGGCTTG | | |  |  |  |
| *Star* | F: | AGAGGCTTTATGAAGAGCTTGTGG | | | 329 | ENSSSCG00000026109 | |
|  | R: | GGTGAGTTTGGTCTTTGAGGGA | | |  |  |  |
| *3β-Hsd* | F: | CCCCTTCAATCGCCACTTCGT | | | 401 | AF232699 | |
|  | R: | CCCAGTCCCCTTGTCCTTCCC | | |  |  |  |
| *Sox3* | F: | TGCCTTGTACCGTTGATGGGGA | | 197 | | ENSSSCG00000022501 |  |
|  | R: | GGAGGAGGGGGGCTGCTTTT | |  |  |  |  |
| *Acta2* | F: | GAGAAGATGACCCAGATTATGTTT | | 493 | | FJ547477 |  |
|  | R: | TGTTGTAGGTGGTCTCATGGAT | |  |  |  |  |
| *Afp* | F: | AGCAAAAGCCACAAATAACAGA | | 208 | | NM_214317 |  |
|  | R: | TTCACACCAAACCATAAAGACC | |  |  |  |  |
| *β-actin-qPCR* | F: | AGGGCAGTAGCATCGCTTTAGT | 118 | | | AY550069 | |
|  | R: | AAGGGGATTGTGATGGCTGA |  |  |  |  |  |
| *Oct4-qPCR* | F: | CAAGCAGTGACTATTCGCAACG | 202 | | | NM_001113060 | |
|  | R: | GAGCCCAGAGGGGTGACAGA |  |  |  |  |  |
| *Sox2-qPCR* | F: | CAACCAGAAGAACAGCCCAGA | 156 | | | NM_001123197 | |
|  | R: | TCCGACAAAAGTTTCCACTCG |  |  |  |  |  |
| *Nanog-qPCR* | F: | TGGATCTGCTTATTCAGGACAGC | 115 | | | NM_001129971 | |
|  | R: | TGCTTCTTGACTGGGACCTTTT |  |  |  |  |  |
| *Lin28-qPCR* | F: | CTGAAGGAGGGTGAGGCTGTG | 198 | | | NM_001123133 | |
|  | R: | GCATTCCTTGGCATGATGGT |  |  |  |  |  |
| *c-Myc-qPCR* | F: | CCCTCCACCAGGAAGGACTAT | 162 | | | NM_001005154 | |
|  | R: | CCTCTGGCGTTCCAAGACATT |  |  |  |  |  |
| *Klft4-qPCR* | F: | AGCCCATCGGTCATCAGTGTTA | 107 | | | NM_001031782 | |
|  | R: | TGCTTGATCTTGGGGCACAT |  |  |  |  |  |
| *Tert-qPCR* | F: | GGAAGATGAAAGTGCAAGACTGC | 171 | | | ENSSSCT00000018636 | |
|  | R: | TGGTCTCCGTGACATAAAAAAAC |  |  |  |  |  |
| *Oct4-DMR* | F: | TTTTGTGGGAGAGTTTAGGGTAG | 180 | | | ENSSSCG00000001393 | |
|  | R: | ATCCCCTCAAAAACTAAACCTC |  |  |  |  |  |
| *Nanog-DMR* | F: | TGAGGTTGGTAGATAGGATTAATTG | 190 | | | ENSSSCG00000004977 | |
|  | R: | AAAATAAAATAAAATAAATCACCCT |  |  |  |  |  |
| *H19-DMR* | F: | GGTTGTGGGTGTGGAGGTAGAAG | 205 | | | AY044827 | |
|  | R: | CACTAAACACCCAACCTTTAACAC |  |  |  |  |  |
