# Supplementary figures and images for: Previously claimed male germline stem cells from porcine testis are actually progenitor Leydig cells
Source: Stem Cell Res Ther. 2018 Jul 18;9:200. doi: 10.1186/s13287-018-0931-0 (PMC6052628; doi:10.1186/s13287-018-0931-0)

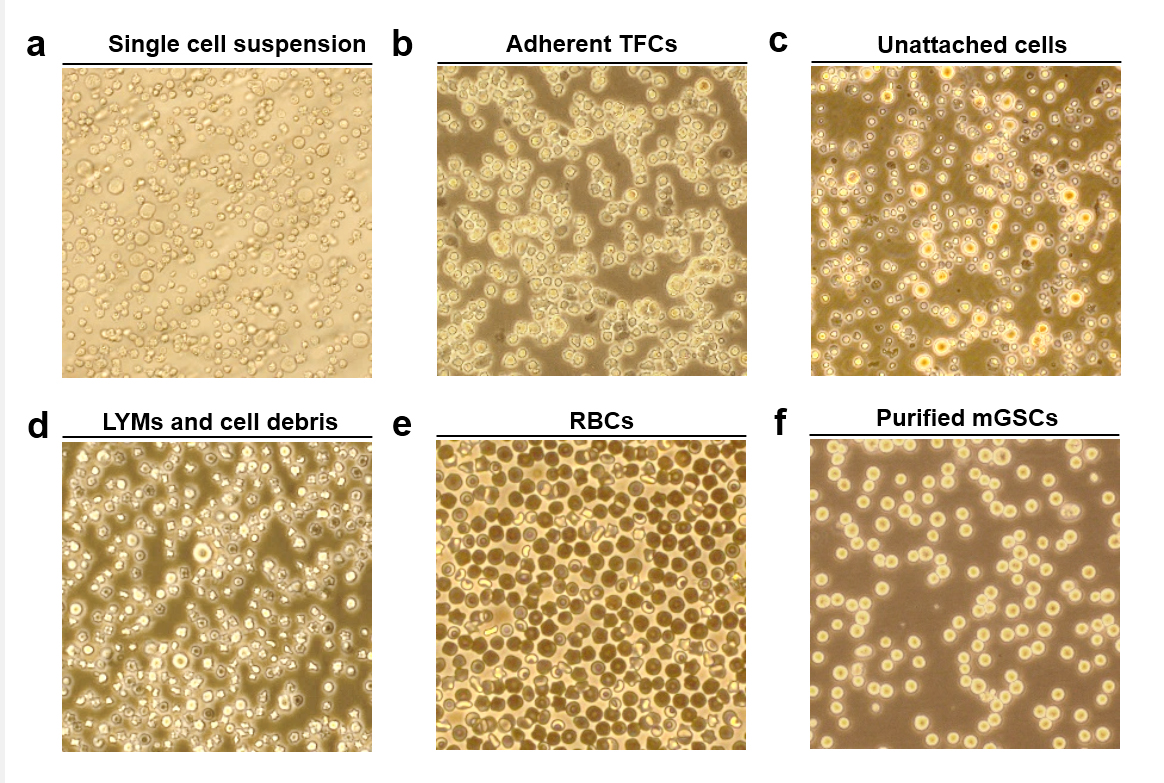

Supplement: Supplementary file 3 — Figure S1. Enrichment of porcine male germline stem cell (mGSCs). (a) The testicular suspension contained several cell types. (b) Testicular fibroblast cells (TFCs) were removed by differential attachment technique. (c) The unattached cells underwent density gradient centrifuging to remove the leukomonocytes (LYMs) and cell debris (d) and red blood cells (RBCs) (e), and to retain mGSCs (f). (JPG 699 kb) [file 13287_2018_931_MOESM3_ESM.jpg]

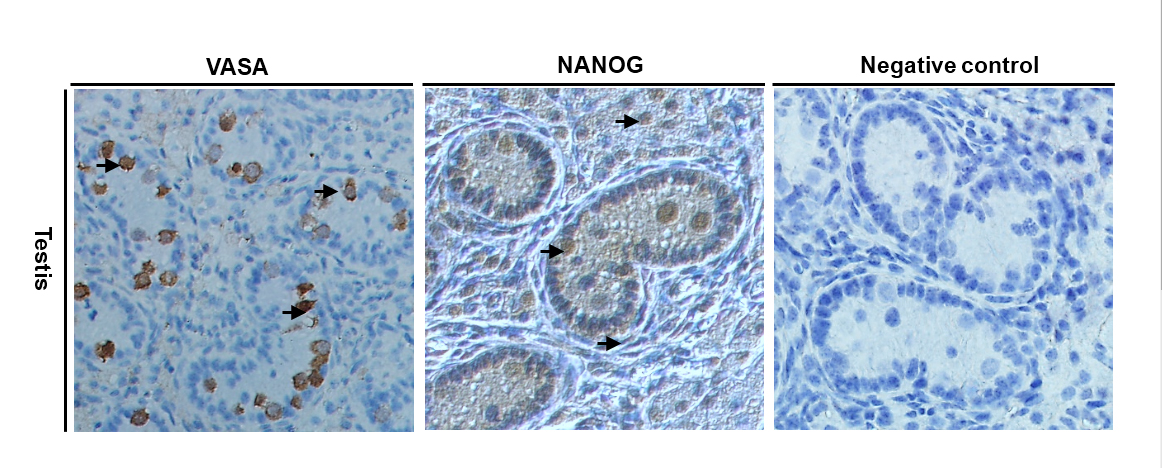

Supplement: Supplementary file 4 — Figure S2. Immunolocalization of VASA and NANOG proteins in the testis of 5-day-old piglet. Immunohistochemistry analysis showed that the reproduction-associated marker VASA localized to the basement membrane of seminiferous tubules, whereas the pluripotent factor NANOG was expressed in the interstitial space and seminiferous tubules. (JPG 442 kb) [file 13287_2018_931_MOESM4_ESM.jpg]

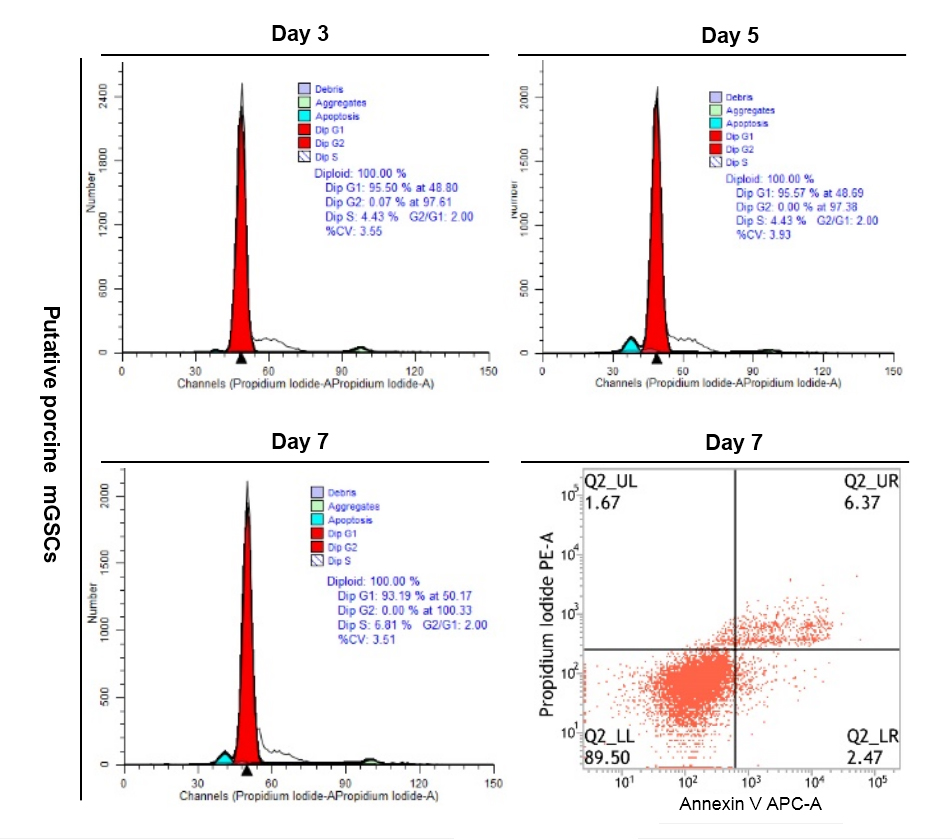

Supplement: Supplementary file 5 — Figure S3. Cell cycle assay of putative porcine male germline stem cell (mGSCs). Cell cycle analyses at cell culture days 3, 5, and 7 revealed that only a small percentage of the putative mGSCs entered the S and G2 cell division phases. Meanwhile, part of putative mGSCs underwent apoptosis detected by analysis of apoptosis at day 7 of cell culture. (JPG 285 kb) [file 13287_2018_931_MOESM5_ESM.jpg]

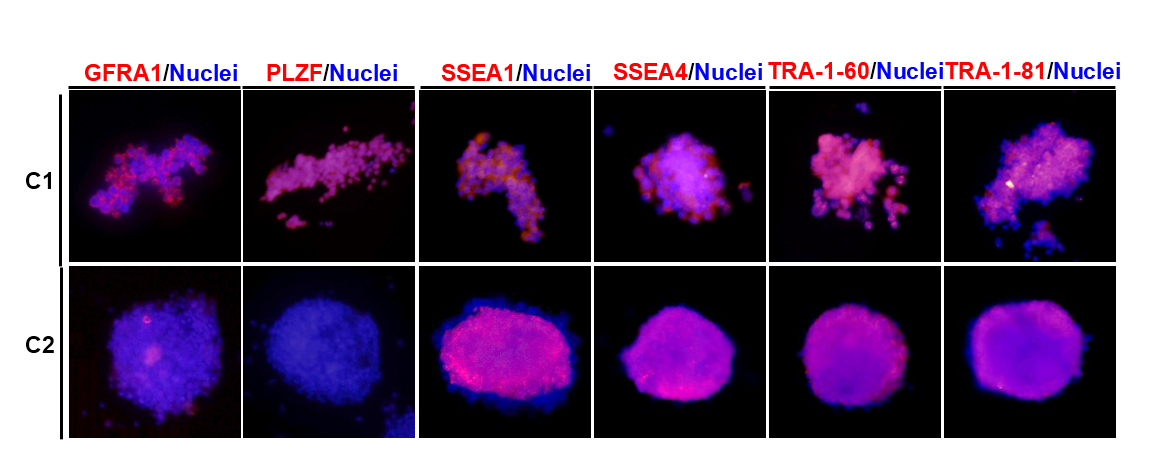

Supplement: Supplementary file 6 — Figure S4. Expression of pluripotency-associated markers and germ cell-specific markers in the C1 and C2 clusters. Immunofluorescent staining showed that both the C1 and C2 clusters expressed the pluripotency-associated markers SSEA1, SSEA4, TRA-1-60, and TRA-1-81. However, the C1 clusters, but not the C2 clusters, expressed the germ cell-specific markers GFRA1 and PLZF. (JPG 249 kb) [file 13287_2018_931_MOESM6_ESM.jpg]

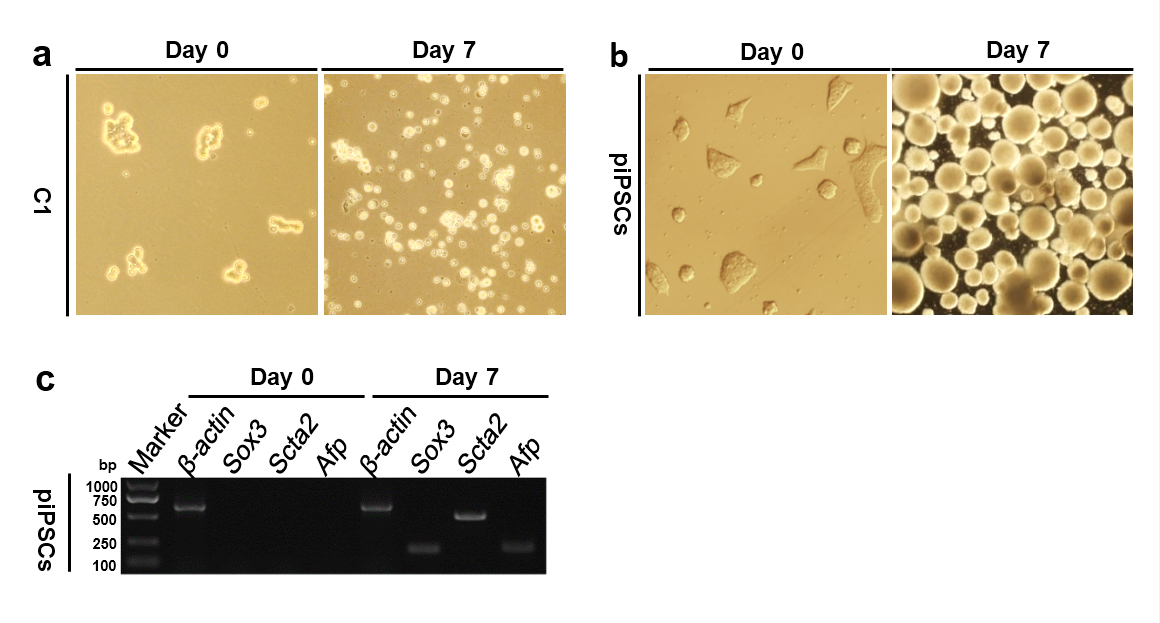

Supplement: Supplementary file 7 — Figure S5. Comparison of pluripotency potential between the C1 clusters and porcine-induced pluripotent stem cells (piPSCs). (a) The C1 clusters and (b) piPSCs were cultured without feeder cells and serum for 7 days to induce embryoid body formation. Embryoid bodies were formed from the piPSCs, but not the C1 clusters, (c) with induction of lineage-specific genes. (JPG 310 kb) [file 13287_2018_931_MOESM7_ESM.jpg]

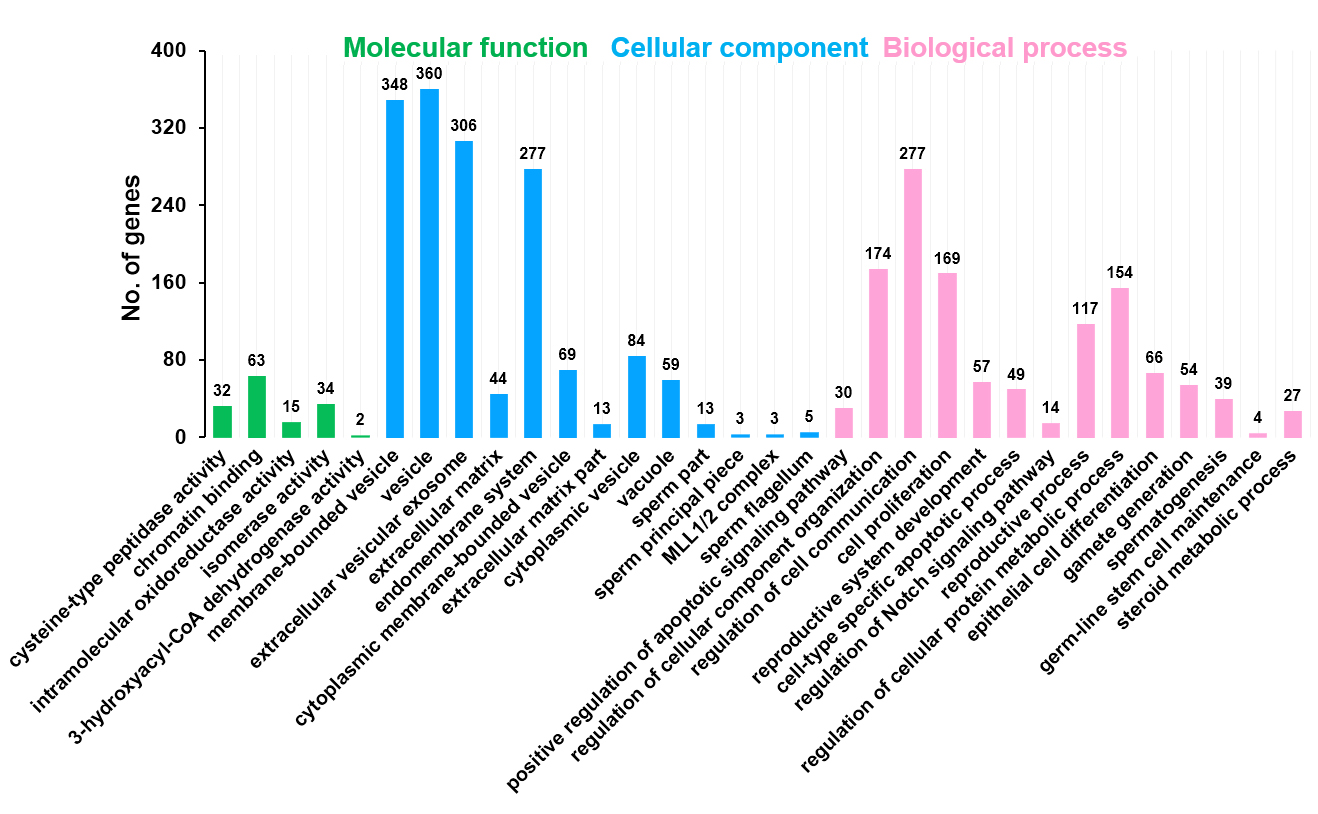

Supplement: Supplementary file 8 — Figure S6. Gene ontology (GO) analysis of the differentially expressed genes between C1 and C2. (JPG 403 kb) [file 13287_2018_931_MOESM8_ESM.jpg]

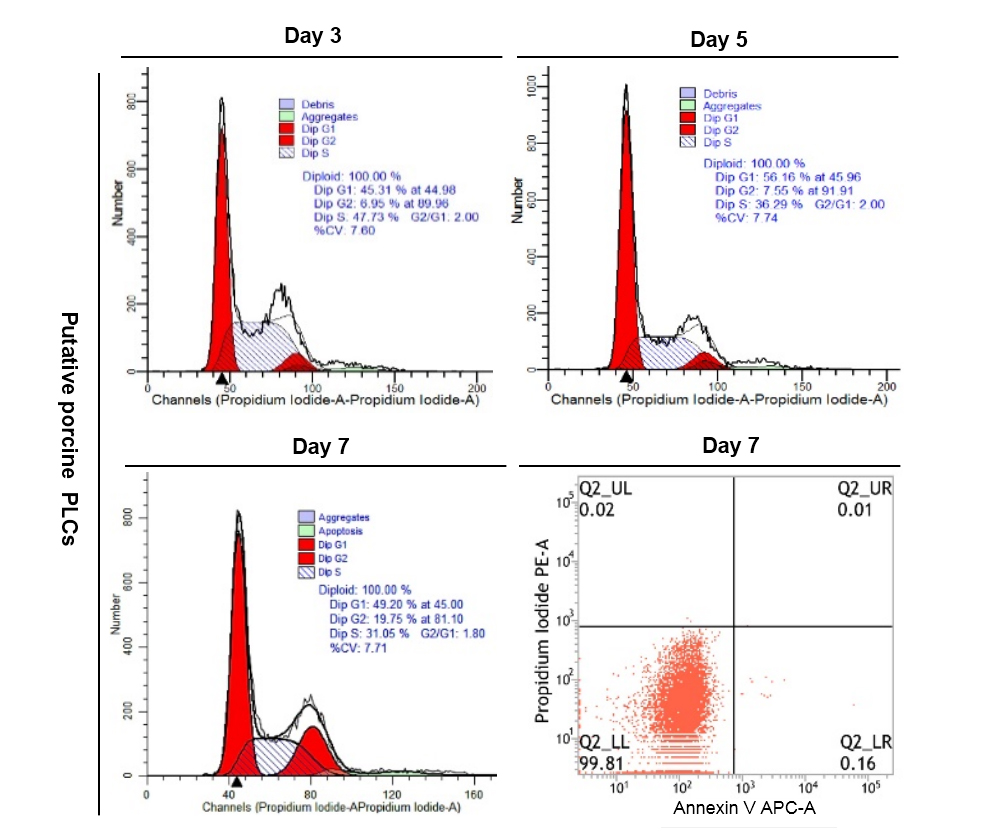

Supplement: Supplementary file 9 — Figure S7. Cell cycle assay of putative porcine progenitor Leydig cells (PLCs). Cell cycle analysis of the PLCs showed that they had rapidly dividing capacities at days 3, 5, and 7 in culture. Analysis of apoptosis showed that PLCs had very low levels of cell death at day 7 in culture (99.81% propidium iodide, annexin V double negative). (JPG 299 kb) [file 13287_2018_931_MOESM9_ESM.jpg]

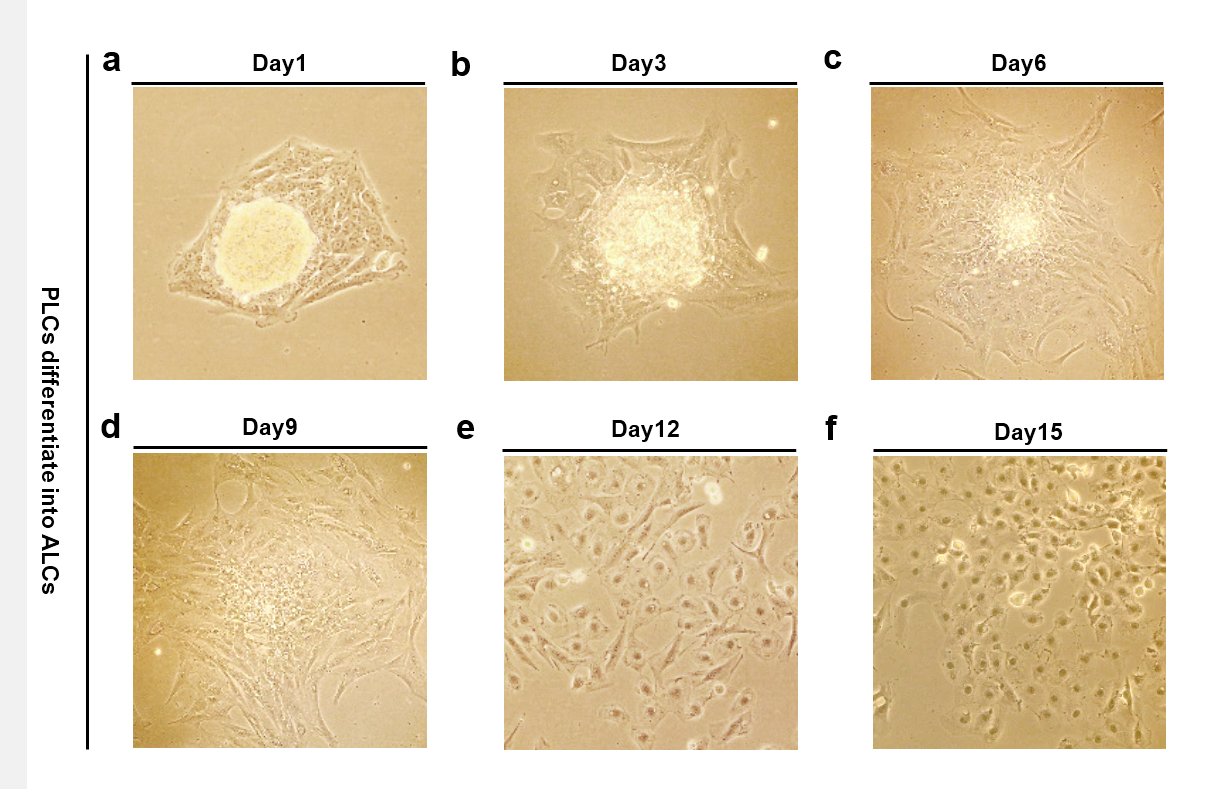

Supplement: Supplementary file 10 — Figure S8. Induction of C2 clusters differentiation into mature adult Leydig cells (ALCs). The C2 clusters began to differentiate into fibroblast cells at day 1 (a), became smaller from days 3 to 6 (b, c), and had disappeared by day 9 (d) The C2 clusters were fully differentiated into ALCs by day 12 (e), with abundant mature ALCs observed by day 15 (f). (JPG 558 kb) [file 13287_2018_931_MOESM10_ESM.jpg]

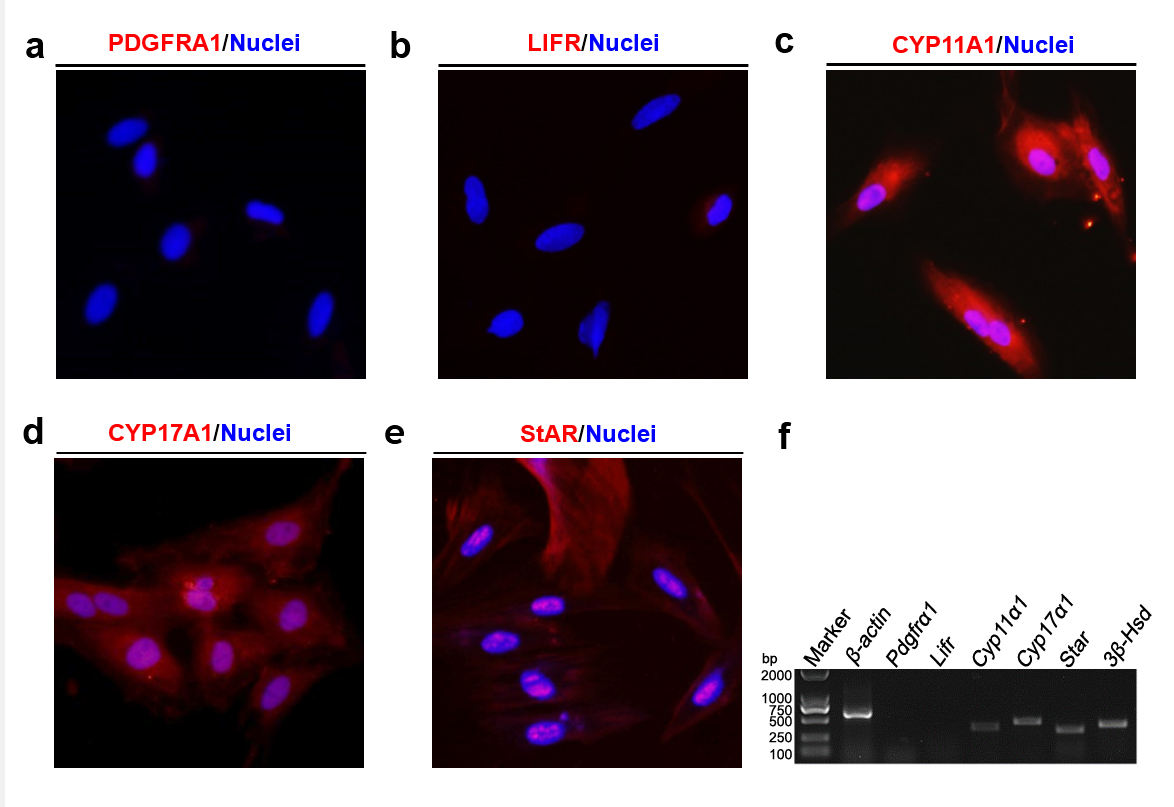

Supplement: Supplementary file 11 — Figure S9. Expression of adult Leydig cell (ALC)-associated markers in induced differentiated C2 clusters. They did not express the PLC markers PDGFRA1 (a) and LIFR (b), but expressed testosterone synthesis enzymes CYP11A1 (c), CYP17A1 (d), and StAR (e), demonstrated at both the protein and mRNA levels (f). (JPG 301 kb) [file 13287_2018_931_MOESM11_ESM.jpg]
